# Abalone peptide increases stress resilience and cost‐free longevity via SKN‐1‐governed transcriptional metabolic reprogramming in *C. elegans*

**DOI:** 10.1111/acel.14046

**Published:** 2023-11-22

**Authors:** Qiangqiang Wang, Liangyi Wang, Ziliang Huang, Yue Xiao, Mao Liu, Huihui Liu, Yi Yu, Ming Liang, Ning Luo, Kunping Li, Ajay Mishra, Zebo Huang

**Affiliations:** ^1^ Institute for Food Nutrition and Human Health, School of Food Science and Engineering, South China University of Technology Guangzhou China; ^2^ Guangdong Province Key Laboratory for Biocosmetics Guangzhou China; ^3^ Center for Bioresources and Drug Discovery, School of Biosciences and Biopharmaceutics, Guangdong Pharmaceutical University Guangzhou China; ^4^ Research and Development Center, Infinitus (China) Company Ltd Guangzhou China; ^5^ Institute of Chinese Medicinal Sciences, Guangdong Pharmaceutical University Guangzhou China; ^6^ European Bioinformatics Institute Cambridge UK

**Keywords:** detoxification, frailty, life span, oxidative stress, peptide, proteotoxicity, resilience, SKN‐1/Nrf

## Abstract

A major goal of healthy aging is to prevent declining resilience and increasing frailty, which are associated with many chronic diseases and deterioration of stress response. Here, we propose a loss‐or‐gain survival model, represented by the ratio of cumulative stress span to life span, to quantify stress resilience at organismal level. As a proof of concept, this is demonstrated by reduced survival resilience in *Caenorhabditis elegans* exposed to exogenous oxidative stress induced by paraquat or with endogenous proteotoxic stress caused by polyglutamine or amyloid‐β aggregation. Based on this, we reveal that a hidden peptide (“cryptide”)—AbaPep#07 (SETYELRK)—derived from abalone hemocyanin not only enhances survival resilience against paraquat‐induced oxidative stress but also rescues proteotoxicity‐mediated behavioral deficits in *C. elegans*, indicating its capacity against stress and neurodegeneration. Interestingly, AbaPep#07 is also found to increase cost‐free longevity and age‐related physical fitness in nematodes. We then demonstrate that AbaPep#07 can promote nuclear localization of SKN‐1/Nrf, but not DAF‐16/FOXO, transcription factor. In contrast to its effects in wild‐type nematodes, AbaPep#07 cannot increase oxidative stress survival and physical motility in loss‐of‐function *skn‐1* mutant, suggesting an SKN‐1/Nrf‐dependent fashion of these effects. Further investigation reveals that AbaPep#07 can induce transcriptional activation of immune defense, lipid metabolism, and metabolic detoxification pathways, including many SKN‐1/Nrf target genes. Together, our findings demonstrate that AbaPep#07 is able to boost stress resilience and reduce behavioral frailty via SKN‐1/Nrf‐governed transcriptional reprogramming, and provide an insight into the health‐promoting potential of antioxidant cryptides as geroprotectors in aging and associated conditions.

## Graphical abstract

We propose a loss‐or‐gain survival model to quantify stress resilience, which is demonstrated in *C. elegans* under oxidative or proteotoxic stress. Based on this, we reveal that a hidden peptide (“cryptide”) derived from abalone can boost stress resilience, reduce behavioral frailty and increase cost‐free longevity via SKN‐1/Nrf‐governed transcriptional activation of immune defense, lipid metabolism and metabolic detoxification pathways. Our findings provide an insight into the health‐promoting potential of cryptides as geroprotectors in aging and associated conditions.

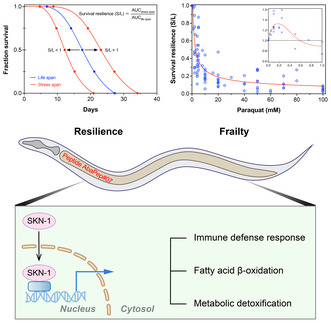

**Abbreviations**
AD: Alzheimer's disease
AUC: area under the curve
Aβ: amyloid‐β peptide
CYP: cytochrome P450
DEG: differentially expressed genes
FC: fold change
FDR: false discovery rate
FUDR: 5‐fluoro‐2′‐deoxyuridine
GC–MS: gas chromatography–mass spectrometry
GFP: green fluorescent protein
GO: gene ontology
GST: glutathione‐S‐transferases
HD: Huntington's disease
IIS: insulin/insulin‐like growth factor signaling
LEA: late embryogenesis abundant
NGM: nematode growth medium
Nrf: nuclear factor erythroid‐2‐related factor
P‐gp: P‐glycoprotein
polyQ: polyglutamine
ROS: reactive oxygen species
S/L: ratio of stress span to life span
SKN‐1: skinhead‐1
UGT: UDP‐glucuronosyltransferases
YFP: yellow fluorescent protein

## 1 INTRODUCTION

A common feature of aging is frailty, which is a state of increased vulnerability across multiple physiological systems due to lifelong accumulation of molecular and cellular deficits as well as metabolic dysregulation, including a progressive decline in the ability to withstand stresses such as excessive reactive oxygen species (ROS), abnormal protein accumulation, and toxicant exposure (Peters et al., 2021; Taylor et al., 2023). Ensued from such cumulative damages are structural alteration and functional deterioration at molecular, cellular and organismal levels, resulting in reduced metabolic and physical fitness. As stress challenges are often persistent throughout the entire life, an organism has to unremittingly respond to such signals and preserve physiological homeostasis to promote adaptation (Huang & Tunnacliffe, 2006; López‐Otín & Kroemer, 2021). For example, ROS is intimately involved in aging and disease during life—homeostatic level of ROS is essential for normal physiological functions but excessive ROS is detrimental (Bazopoulou et al., 2019). Another example is protein aggregation, which involves aberrant accumulation of aggregated proteins during aging and stress. Many of these proteins are, however, physiologically important at normal levels but become pathologically altered upon disruption of protein homeostasis (proteostasis) by intrinsic and extrinsic stress conditions, leading to aging and disease (Santra et al., 2019; Walther et al., 2015).

The ability of an organism to maintain homeodynamics or quickly reach a new homeostasis when experiencing deleterious perturbations is known as resilience, the loss of which manifests much earlier than the onset of frailty (Borras et al., 2020; Ferrucci et al., 2020). In general, the homeostatic resilience of an aging organism degenerates while its vulnerability to metabolic and stress damages increases, leading to increased frailty and eventual mortality (Ferrucci et al., 2020; López‐Otín & Kroemer, 2021). Therefore, strategies that help to orchestrate homeostatic resilience represent a promising approach to enhance damage control during aging and stress. In this regard, Nature has its own wisdom. In response to extreme desiccation, for example, some organisms enter anhydrobiosis (“life without water”), a cryptobiotic state (“hidden life”) of reversible homeostasis characterized by virtually no metabolic activity, avoiding stress‐triggered deterioration (Huang & Tunnacliffe, 2006). Anhydrobiotic organisms can resume normal life from such suspended animation (in theory an unlimited “life”)—almost without cost to their normal life cycle and life span—when conditions become favorable again (Huang & Tunnacliffe, 2006; Kaczmarek et al., 2019). The mechanisms that govern anhydrobiosis implicate, among others, detoxification associated proteins and intrinsically disordered proteins, including late embryogenesis abundant (LEA) proteins in particular (Boothby et al., 2017; Browne et al., 2002; Fu et al., 2020). Nevertheless, whether and how such strategies (e.g., extremely low metabolism) and underlying molecules (e.g., LEA proteins) can be used in other systems or as potential therapeutics are still underexplored and merit further investigation.

Another hidden wisdom from Nature is “cryptides” (cryptic peptides—short amino acid sequences encrypted in natural proteins) and “cryptome” (a repertoire of cryptic peptides)—many such cryptides are inactive when “hidden” in proteins but exert functions upon release (Iavarone et al., 2018; Xiao et al., 2022; Zhang et al., 2021). For example, ~2500 peptides are identified in enzymatically prepared oyster protein hydrolysate, demonstrating the release of a large repertoire of cryptides (Zhang et al., 2021). The peptides are found to increase serum testosterone content in mice and gene level of *Caenorhabditis elegans* LET‐767, a homolog of human 17‐β‐hydroxysteroid dehydrogenases that have important roles in androgen and estrogen metabolism (Zhang et al., 2021). Another example is the proteolytically generated sea cucumber peptide fractions, which also contain >1000 peptides in total and are shown to promote oxidative stress survival, decrease age pigments, and extend life span in *C. elegans* (Guo et al., 2020; Lu et al., 2021). Interestingly, LEA‐derived peptides can function like LEA proteins in bacteria and *C. elegans* to improve proteostasis and desiccation resistance (Hibshman & Goldstein, 2021). Overall, peptides are increasingly reported to have geroprotective properties, including stress resistance, life span extension, and disease alleviation (Muttenthaler et al., 2021; Xiao et al., 2022). However, the vast majority of cryptides have thus far remained largely elusive, lacking clear structural and functional information.

As a rich source of chemically diverse and biologically active molecules, marine organisms have drawn significant attention for biomolecular discovery (Ovchinnikova, 2021; Xiao et al., 2022). One of the most valued marine organisms is abalone, the gastropod belonging to the genus *Haliotis* which has been used for centuries across the globe and widely considered a health‐promoting seafood. For example, various abalones are traditionally used in tonic remedies or to treat ailments, including antifatigue and antistress applications, in East Asia (Shi et al., 2020), while red abalone (*Haliotis rufescens*) is a culturally iconic gastropod native to the west coast of North America (Swezey et al., 2020). Transcriptional studies suggest that disk abalone (*Haliotis discus discus*) can use antioxidant and immune defense mechanisms to tackle physical stresses, including alterations in temperature, salinity, and oxygen levels (De Zoysa et al., 2009). Abalone is also a good source of proteins, which comprises ~50% (dry weight) of its edible part (Boamah et al., 2020; Shi et al., 2020). Interestingly, abalone‐derived peptides are shown to suppress photoaging of keratinocyte cells (Chen et al., 2019) and vasculogenesis of tumor cells (Gong et al., 2019). Recently, two precursors of tachykinins, a class of neuropeptides with rapid response to injury, and two tachykinin receptor isoforms are also found to play a potential role in regulating lipid metabolism in abalone (Lee et al., 2022).

In this study, we first proposed a loss‐or‐gain survival model in *C. elegans* to quantify stress resilience at organismal level and tested the effect of an abalone protein hydrolysate on the survival resilience of *C. elegans* against oxidative stress. A novel antioxidant octapeptide identified from the hydrolysate was then subjected to geroprotective studies, including stress resistance, proteotoxicity, and life span assays, as well as to growth and reproduction examinations. Further investigations were focused on the stress and life span related transcription factors SKN‐1/Nrf and DAF‐16/FOXO. Finally, transcriptomic analysis revealed an association of the abalone peptide with immune defense, lipid metabolism, and metabolic detoxification pathways in *C. elegans*.

## 2 MATERIALS AND METHODS

### 2.1 Bacterial strains

*Escherichia coli* OP50 and NA22 were cultured in Luria Bertani (LB) medium overnight at 37°C. *E. coli* OP50 was spread on nematode growth medium (NGM) plates and allowed to grow overnight before use, while *E. coli* NA22 was concentrated 10‐fold by centrifugation at 8000 × *g* and stored at 4°C. All bacterial preparations were used within 2 weeks.

### 2.2 Nematode strains and maintenance

The following *C. elegans* strains used for this study were obtained from *Caenorhabditis* Genetics Center (University of Minnesota): Bristol N2, AM140 [*rmIs132*(*unc‐54p::Q35::YFP*)], GMC101 [*dvIs100*(*unc‐54p::Abeta‐1‐42::unc‐54 3’‐UTR* + *mtl‐2p::GFP*], LG326 [*skn‐1*(*zu169*)*IV; Is007*(*skn‐1::GFP*)], GR1352 {*daf‐16*(*mgDf47*)*I*; *xrIs87*[*daf‐16*(*alpha*)::*GFP*::*daf‐16B* + *rol‐6*(*su1006*)]}, EU1 {*skn‐1*(*zu67*)*IV*/*nT1*[*unc‐*?(*n754*)*let‐*?]}, and KU25 [*pmk‐1*(*km25*)*IV*]. Nematodes were grown and maintained on NGM agar plates seeded with *E. coli* OP50 as food at 20°C unless otherwise noted, while experiments in liquid culture were performed in S medium with *E. coli* NA22 as food. Synchronization of nematodes was performed using the standard alkaline hypochlorite method.

### 2.3 Peptides

Fresh abalone (*Haliotis diversicolor*) were purchased from Huangsha Seafood Market, Guangzhou, China. Abalone protein hydrolysate was prepared by sequential trypsin–papain enzymatic hydrolysis and fractionated by successive ultrafiltration and gel filtration, and sequences of peptides in antioxidant fractions were identified by liquid chromatography–tandem mass spectrometry with a reverse‐phase nanocolumn (RP‐nano‐LC–MS/MS) coupled with database‐assisted peptide sequencing as previously described (Guo et al., 2020). After in silico analysis of the identified sequences for antioxidant potential using BIOPEP‐UWM database (http://www.uwm.edu.pl/biochemia/index.php/pl/biopep), peptides of interest were synthesized and tested for antioxidant activity using paraquat survival assay in *C. elegans* (Table S3) as detailed previously (Guo et al., 2020; Lu et al., 2021). The peptide AbaPep#07 (SETYELRK) was synthesized by Shanghai Top‐Peptide Biotechnology Co. Ltd (Shanghai, China) with >95% purity as previously (Lu et al., 2021) and used for further studies.

### 2.4 Oxidative stress survival assay

The survival of *C. elegans* under increased oxidative stress was assessed as previously performed (Guo et al., 2020) with modifications. Briefly, ~20 synchronized L4 nematodes were transferred into the wells of a 96‐well plate (>100 nematodes for each treatment) with 100 μL of S medium containing test samples, *E. coli* NA22 (OD_570nm_ of ~0.5) and 75 μg/mL FUDR. After incubation for 24 h, the nematodes were exposed to 50 mM paraquat. The live and dead nematodes were scored microscopically based on their movement and pharyngeal pumping every 12 h until all dead. Data were presented in Kaplan–Meier survival curves, and log‐rank (Mantel‐Cox) method was used to determine the significance difference. To investigate the overall resilience of *C. elegans* to stress, the survival resilience was expressed as the ratio of stress span to life span (S/L), which was quantitated by the area under the survival curve (AUC) of the stress‐exposed animals over that of the control animals under normal condition (Equation 1):

(1) $$\text{Survival resilience}\;\left(S/L\right)={\mathrm{AUC}}_{\text{stress span}}/{\mathrm{AUC}}_{\text{life span}}$$

More specifically, to assess the effect of abalone peptides on the survival resilience of *C. elegans* against oxidative stress, the above equation of survival resilience was adjusted as follows (Equation 2):

(2) $$\text{Survival resilience}={\mathrm{AUC}}_{\text{treatment}}/{\mathrm{AUC}}_{\text{life span}}$$

where AUC_treatment_ is the survival AUC of the paraquat‐exposed nematodes with or without peptide pretreatment, and AUC_life span_ is the AUC of the control nematodes with neither paraquat exposure nor peptide treatment (i.e., normal life span).

### 2.5 Assessment of polyQ‐ and Aβ‐mediated behavioral dysfunction

Approximately 200 synchronized L4 nematodes were transferred to each well of a 48‐well plate with 0.5 mL of S medium containing test sample, *E. coli* NA22 (OD_570 nm_ of ~0.5), 75 μg/mL FUDR, and 100 μg/mL ampicillin (3 replicate wells for each treatment). The plates were placed in a shaking incubator at 20°C (recorded as Day 0) for further culture. For the AM140 strain, the plates were incubated at 20°C for 5 or 10 days. For the GMC101 model, the plates were incubated at 20°C for ~12 h, and then, the temperature was upshifted to 25°C (to induce Aβ expression) for a further culture period of 3 or 5 days. Then, the nematodes in each condition were harvested, respectively, at the indicated times into 1.5 mL microcentrifuge tubes and washed 3 times with M9 buffer prior to test. To measure proteotoxicity‐associated directional shifts and paralysis rate, ~30 nematodes from each condition were transferred to a food‐free 3.5 cm NGM plate and allowed to acclimatize for 10 min. Tracking videos were subsequently recorded for 30 s by using a stereomicroscope at 4× magnification with a CMOS camera (Chongqing Optical Instrument Co., Ltd., Chongqing, China) and analyzed by the Movement Tracker software as described (Mouchiroud et al., 2016). The parameters of “Direction” and “Fraction Paralyzed” in the movement data were used to calculate average number of directional shifts and fraction of paralyzed nematodes, respectively. The readouts of directional shifts of nematodes in each treatment were compared with the average number in the control and thus normalized as a direction rescue index, which was calculated as follows (Equation 3):

(3) $$\text{Direction rescue index}={\text{Nematode}}_{\text{below}-\text{mean}}/{\text{Nematode}}_{\text{total}}$$

where, in a treatment group, Nematode_below‐mean_ is the number of nematodes whose directional shifts are less than the average number of directional shifts in the untreated control group (i.e., “control mean”), and Nematode_total_ is the total number of nematodes in the same treatment group. If more than 80% of the instantaneous speeds measured during the tracking were less than 0.015 mm/s, the nematodes were regarded as paralyzed (Ramot et al., 2008).

### 2.6 Quantification of polyQ aggregation

PolyQ aggregation assay was performed as previously (Zhang et al., 2012) using *C. elegans* AM140, a strain expressing Q35::YFP fusion protein in body wall muscle cells. In brief, synchronized L4 nematodes were incubated and collected at 20°C as above. The nematodes were immobilized with 20 mM sodium azide on 2% agarose pads on glass slides. Fluorescent images were taken using an Mshot MF31‐LED fluorescence microscope (Guangzhou Micro‐shot Technology Co. Ltd.) fitted with a 10× objective lens and FITC filter. The number of Q35::YFP aggregates was counted manually (>30 nematodes for each treatment).

### 2.7 Life span assay

Strict temperature, food abundance, and population density were controlled for life span assays, which were conducted in liquid culture as previously described (Zhang et al., 2012). Concisely, synchronized L4 nematodes were transferred into 96‐well plates (~20 nematodes/well, >100 nematodes for each treatment) with the addition of test sample, *E. coli* NA22 (OD_570 nm_ of ~0.65), 75 μg/mL FUDR and 100 μg/mL ampicillin. The plates were sealed with parafilm and then incubated in a 20°C shaker at 120 rpm. The live and dead nematodes were counted microscopically every 2 days based on their movement and pharyngeal pumping until all dead. Data were presented in Kaplan–Meier survival curves, and log‐rank (Mantel‐Cox) method was used to determine the significant difference. All life span experiments were repeated independently at least three times.

### 2.8 Fecundity assay

Synchronized wild‐type L4 nematodes were individually placed on 3.5 cm NGM plates seeded with OP50 in the presence or absence of abalone peptide (1 nematode per plate, 15 nematodes per treatment) and allowed to lay eggs for 24 h. The parent nematodes were transferred to freshly seeded plates every day until egg laying ceased. The plates containing eggs were incubated at 20°C for 48 h, and the number of progeny was counted for brood size determination.

### 2.9 Size and motility analysis

Wild‐type N2 nematodes with or without abalone peptide treatment in 48‐well plates were collected on Day 1 and Day 10 as indicated. For measurement of *C. elegans* movements on solid agar media, motility videos were recorded and analyzed by the Movement Tracker software as described above. The parameters of “SizeAverage,” “Direction,” and “SpeedMean” in the output data were used to calculate body size, number of directional shifts and crawling speed, respectively (Mouchiroud et al., 2016). The normalized body size was expressed as a ratio of the body size mean in each treatment to that of the control nematodes. For measurement of swimming speed and thrashing rate in liquid media, approximately 10 nematodes for each treatment were placed in a drop of M9 buffer and their swimming movements were recorded for 30 s after a 10 s recovery period. The video files were then processed and analyzed using wrMTrck plugin in ImageJ software as described (Nussbaum‐Krammer et al., 2015), and the parameters of “avgSpeed” and “BBPS” (body bends per second) in the output data were used to measure swimming speed and thrashing frequency, respectively. The results of size and motility analysis are presented as a radar chart for multiple metrics using OriginPro software as previously described (Wang et al., 2021). To assess the effect of abalone peptide on the overall physical health of *C. elegans*, the fitness landscape represented by multiple distinct metrics was integrated into a single “total fitness,” which was an average value of all ratios of the treated nematodes to the control nematodes for each individual fitness metrics and calculated as follows (Equation 4):

(4) $$\text{Total fitness}=\left({\text{M1}}_{t}/{\text{M1}}_{c}+{\text{M2}}_{t}/{\text{M2}}_{c}+{\text{M3}}_{t}/{\text{M3}}_{c}+{\text{M4}}_{t}/{\text{M4}}_{c}+{\text{M5}}_{t}/{\text{M5}}_{c}\right)/5$$

where M_t_ and M_c_ represent a fitness metric of treated and control nematodes, respectively, while a number indicates a specific fitness metric.

### 2.10 Nuclear localization assay of SKN‐1 and DAF‐16

SKN‐1 nuclear localization was analyzed using the transgenic LG326 nematode that expresses a fusion protein of SKN‐1 tagged with a GFP reporter (Bishop & Guarente, 2007). DAF‐16 nuclear localization assay was performed using the transgenic GR1352 strain expressing DAF‐16::GFP fusion protein (Oh et al., 2005). Briefly, synchronized L1 larvae in each experiment were incubated with or without abalone peptide to L4 stage at 20°C. The nematodes were then harvested and transferred into 24‐well plates, immobilized with 2 mM sodium azide, and covered with 12 mm round coverslips for image acquisition as previously described (Zhang et al., 2016). Fluorescent images were captured by using an ImageXpress Micro System (Molecular Devices) with a FITC filter and 20× magnification. For quantification, the LG326 nematodes were categorized into three groups (i.e., “low,” “medium,” and “high”) depending on the levels of SKN‐1::GFP nuclear accumulation in the intestinal nuclei as described (An & Blackwell, 2003). “Low” represents that SKN‐1::GFP nuclear accumulation is barely detectable through the intestine, “medium” refers to the nematodes in which SKN‐1::GFP nuclear accumulation is partially present in intestine, and “high” indicates that a strong SKN‐1::GFP signal is observed in almost all intestinal nuclei. Similarly, the GR1352 nematodes in each treatment were classified as “cytoplasmic,” “intermediate,” and “nuclear” groups based on DAF‐16::GFP subcellular distribution as previously described (Oh et al., 2005). “Cytoplasmic” indicates that DAF‐16::GFP signal is diffusely and predominantly localized in the cytoplasm, “intermediate” represents that a small amount of DAF‐16::GFP is distributed in the nuclei but most DAF‐16::GFP is in cytoplasm in some tissues, and “nuclear” means that DAF‐16::GFP localization is present in the nuclei throughout the entire body. Approximately 30 nematodes per condition in each experiment were randomly selected to score subcellular localization of GFP‐fused proteins, and the percentage of nematodes in each category was calculated. All experiments were repeated independently at least two times with similar results.

### 2.11 RNA sequencing

Wild‐type N2 nematodes with or without abalone peptide treatment from L4 were collected at Day 10 of adulthood, washed with M9 buffer at least 3 times, and immediately flash‐frozen in liquid nitrogen. Pellets of nematodes were stored at −80°C and then sent to BGI Genomics (Shenzhen, China) for RNA sequencing (RNA‐seq) analysis. Briefly, total RNA was extracted from whole nematodes using TRIzol kit (Invitrogen) according to the manufacturer's instruction. Then, RNA quality and quantity were evaluated by using a Nanodrop and Agilent 2100 bioanalyzer (Agilent Technologies). The mRNA was purified from the total RNA using oligo(dT)‐attached magnetic beads and sheared into small fragments. Then, cDNA was generated using random hexamer‐primed reverse transcription, amplified by PCR, purified by AMPure XP beads, and qualified using the Agilent Technologies 2100 bioanalyzer. Double stranded PCR products were heat‐denatured and circularized, and amplified with phi29 polymerase (Thermo Fisher Scientific) to make DNA nanoballs (DNBs), which were subsequently loaded into patterned nanoarray and sequenced on the BGIseq500 platform as previously described (Zhang et al., 2022).

### 2.12 RNA‐seq data analysis

The raw sequencing data were subjected to quality control with SOAPnuke (v1.5.2) software (Li et al., 2008). The clean reads were aligned to the *C. elegans* reference genome WBcel235 using HISAT2 (v2.0.4) (Kim et al., 2015). Ericscript (v0.5.5) was subsequently used to detect fusion genes (Benelli et al., 2012), and rMATS (v3.2.5) was used to identify differential splicing genes (Shen et al., 2014). Bowtie2 (v2.2.5) was applied to align the clean reads to the reference coding gene set (Langmead & Salzberg, 2012), and the expression level of genes was calculated by RSEM (v1.2.12) (Li & Dewey, 2011). Differential expression analysis was performed using PossionDis (Audic & Claverie, 1997) with a significance threshold of false discovery rate (FDR) ≤0.05 and fold change (FC) ≥2 (i.e., |log_2_(fold change)| ≥1), and log‐transformed data were used in subsequent analyses. Gene ontology (GO) enrichment analysis of annotated differentially expressed genes (DEG) was performed by Phyper function in R software based on the Hypergeometric test. The significant levels of GO terms were corrected by *Q*‐value with a rigorous threshold (*Q*‐value ≤0.05) by Bonferroni. Protein functional classifications of DEG were based on the InterPro and GO annotations (Wu et al., 2019). To identify genes that may be direct targets of SKN‐1, 1.5 kb upstream of each DEG was analyzed for the presence of consensus SKN‐1 binding motifs TTDTCATC or WWTRTCAT (D = A/G/T, W = A/T, R = A/G) as previously described (Ewald et al., 2015) by using the program matrix‐scan in Regulatory Sequence Analysis Tools (RSAT) (Turatsinze et al., 2008). Graphs were generated using WebLogo (Crooks et al., 2004).

### 2.13 Quantitative real‐time PCR

Total RNA of Day 10 nematodes treated as above was extracted using TransZol Up Kit (TransGen Biotech) and reverse‐transcribed with EasyScript® All‐in‐One First‐Strand cDNA Synthesis Kit (TransGen Biotech) according to the manufacturer's instructions. Quantitative real‐time PCR was carried out on a StepOnePlus Real‐Time PCR Instrument (Applied Biosystems) using PerfectStart^®^ Green qPCR SuperMix Kit (TransGen Biotech) with *act‐1* as a reference gene. All reactions were performed in three technical replicates from three biological repeats. The primers of gene transcripts used for the PCR analysis are listed in Table S9.

### 2.14 Fatty acid profiling analysis by GC–MS

Wild‐type N2 nematodes were treated with or without abalone peptide and collected as above on Day 10 of adulthood. Pellets of nematodes were suspended in 0.5 mL of 2:1 chloroform: methanol (v/v) solution and sonicated on ice. Samples were centrifuged at 14000 rpm for 5 min, and 300 μL of the bottom organic layer was transferred to a new microcentrifuge tube and mixed with an equal volume of ultrapure water. After vortexing for 1 min, the mixture was centrifuged again at 14000 rpm for 5 min. Then, 50 μL of the bottom organic layer, which contained the extracted lipids, was derivatized by 40 μL of Meth‐Prep II reagent (Grace) for 1 h at room temperature to generate fatty acid methyl esters (FAME). Fatty acid profiling analysis was carried out on GC–MS (Agilent 7890B GC/5977B MSD) equipped with a DB‐23 column (60 m × 0.25 mm × 0.15 μm) as previously described (Li et al., 2018). The fatty acids were identified by comparison with FAME Mix C4‐C24 standard (Supelco), and their relative abundance was determined by dividing each fatty acid by the total fatty acid pool (Anderson et al., 2019).

### 2.15 Statistical analysis

Statistical analysis was performed by GraphPad Prism (version 9.1.1 for macOS, GraphPad Software). Statistical significance was assessed by two‐tailed unpaired *t* test between two groups and by one‐way analysis of variance (ANOVA) and Dunnett's multiple comparisons test among multiple groups. Survival data of *C. elegans* were plotted as Kaplan–Meier curves by GraphPad Prism and analyzed using the log‐rank test by SPSS software (version 24.0 for macOS, IBM Corp.). Radar charts of motility analysis were created by OriginPro (version 9.8.5.204 for Windows, OriginLab Corp.,). Transcriptomic data were analyzed using the BGI Dr. Tom online work platform (https://biosys.bgi.com, accessed on September 1, 2022).

## 3 RESULTS

### 3.1 Abalone peptide promotes survival resilience against increased oxidative stress

The overall resilience of an organism will by nature decrease with increasing age and stress, and the resilient homeodynamics may collapse when aging or stress approaches a tipping point, ultimately leading to death. For example, the ability of *C. elegans* to resist increased oxidative stress induced by paraquat, a potent ROS generator widely used in multiple model organisms, has been shown to decrease with age (Vanfleteren, 1993; Yanase et al., 2002). To investigate the overall resilience of resistance to increasing oxidative stress, we propose a conceptual loss‐or‐gain model based on collective survival competence of *C. elegans*, in which the variability of resilience is defined as the difference in cumulative survival between stress‐exposed and normal populations across their entire life. The survival resilience is therefore expressed as a ratio of stress span to life span (S/L), which is calculated by dividing the area under the curve (AUC) of lifelong survival curve of the stress‐exposed population with that of the control population under normal condition (Equation 1). An S/L ratio of <1, as characterized by a shift of the survival curve to the left, indicates a loss of survival resilience, while a ratio of >1—a shift of the curve toward the right—represents a gain of survival resilience (Figure S1).

**FIGURE 1:**
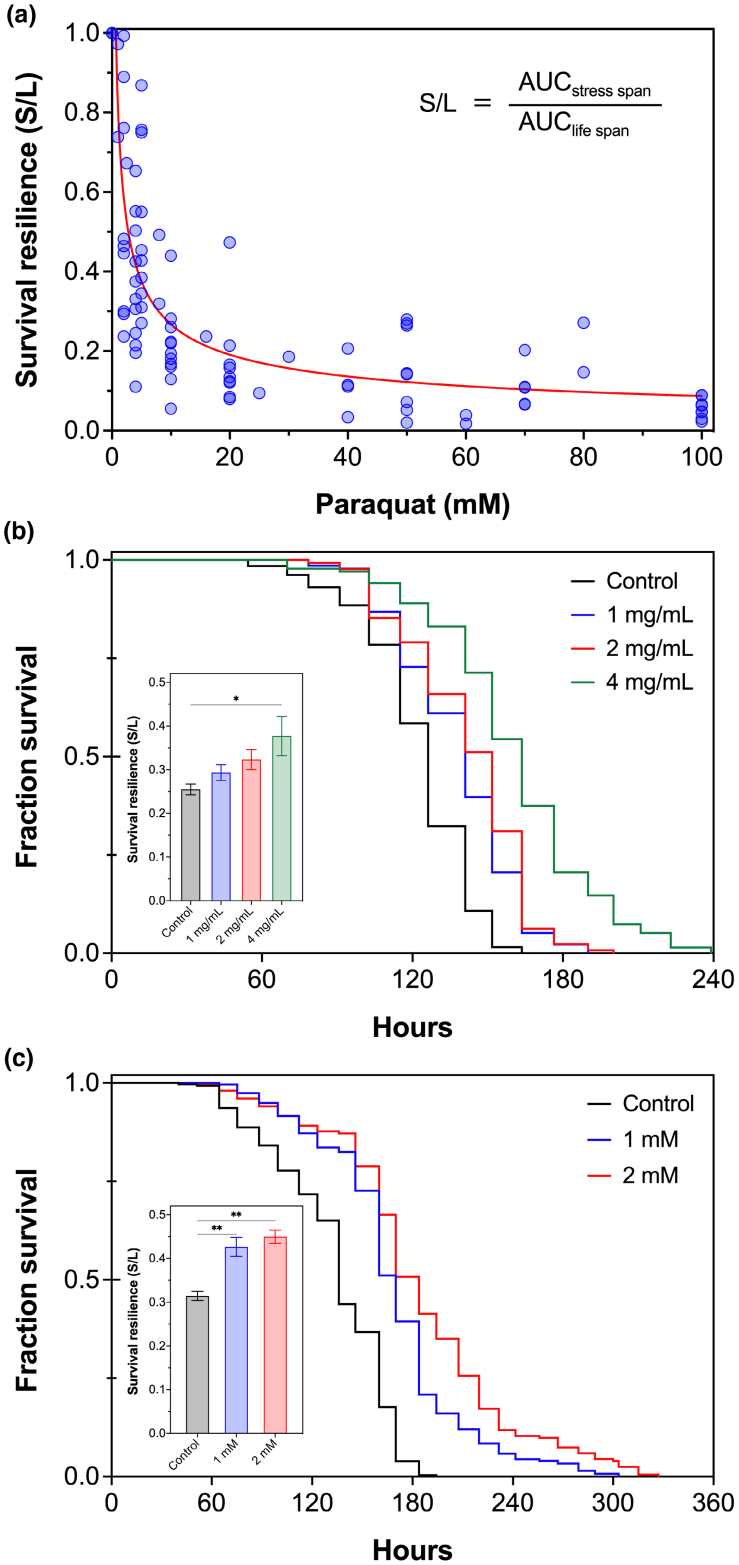
Abalone peptide increases survival resilience of *C. elegans* against oxidative stress. (a) Variability of organismal survival resilience under increasing oxidative stress induced by paraquat. Life span and paraquat survival data of wild‐type nematodes collected from literature (>90 publications; see Table S1) are aggregated and analyzed for survival resilience (Figure S1), which shows that high doses (1–100 mM) decrease the survival resilience while lower doses (see also Figure S2) maintain or even increase the resilience. (b,c) Effect of abalone protein hydrolysate (b) and peptide AbaPep#07 (c) on organismal survival resilience against oxidative stress. Wild‐type L4 nematodes were pretreated with protein hydrolysate or peptide AbaPep#07 for 24 h at 20°C and then exposed to 50 mM paraquat. Live and dead nematodes were scored every 12 h until all dead. Representative results are presented as Kaplan–Meier survival curves. Insets show increase of survival resilience of paraquat‐exposed nematodes by the protein hydrolysate or peptide according to Equation 2. Data are mean ± SEM of three independent experiments. *p < 0.05; **p < 0.01. See Table S2 for further information.

To analyze the impact of increasing oxidative stress on the survival resilience of *C. elegans*, specifically in response to different paraquat doses, we collected and aggregated life span and paraquat survival data in wild‐type N2 nematodes from >90 publications (Table S1). Based on the S/L ratio calculated for each publication, the aggregated data clearly show that paraquat increasingly reduces the survival resilience (S/L < 1) of wild‐type *C. elegans* at doses of 1–100 mM (Figure 1a). Interestingly, paraquat can also maintain or even promote the survival resilience (S/L ≥ 1) of *C. elegans* at very low concentrations (0.01–0.6 mM) (Figure S2 and Table S1), reminiscent of a hormetic response of *C. elegans* to paraquat. Of note, the survival resilience is relatively stable for *C. elegans* exposed to 20–50 mM of paraquat. A lethal oxidative stress is generated when paraquat is persistently applied to *C. elegans* at this level of doses, which are often used for tolerance test against oxidative stress. For example, 50 mM of paraquat has been used in survival assays in a number of oxidative stress studies (see examples listed in Table S1), which can be used in the discovery of strategies to prevent oxidative damages.

We have recently prepared a number of bioactive protein hydrolysates and peptide‐rich fractions from different marine organisms (e.g., Guo et al., 2020; Zhang et al., 2021). Here, we reveal that an abalone protein hydrolysate enzymatically prepared from *Haliotis diversicolor* was able to substantially increase the cumulative survival, as well as survival resilience, of *C. elegans* under oxidative stress induced by persistent treatment with 50 mM of paraquat (Figure 1b and Table S2; Equation 2). Then, AbaPep#07 (SETYELRK), a novel peptide derived from the protein hydrolysate (Table S3), was also shown to significantly promote *C. elegans* survival under paraquat‐induced oxidative stress (Figure 1c and Table S2). It is worth mentioning that, unlike AbaPep#07—the octapeptide fragment “hidden” in abalone hemocyanin as revealed by BLAST analysis (data not shown), several other abalone and sea cucumber peptides tested were unable to promote survival of *C. elegans* under the same oxidative stress (Figure S3). Interestingly, AbaPep#07 was also found to increase the survival resilience of *C. elegans* under paraquat stress, with the S/L ratio being increased from ~0.31 in the control nematodes (treated with paraquat alone) to ~0.45 in the paraquat‐exposed nematodes pretreated with the peptide, an ~45% increase (Figure 1c inset; Equation 2), demonstrating strong antioxidant and resilience‐promoting effects of the abalone peptide.

### 3.2 Abalone peptide reduces behavioral dysfunction induced by proteotoxic stress

Maintenance of proteostasis is required for organisms to integrate environmental and physiological signals and provide a coordinated global response to protect their survival resilience. However, intrinsic and extrinsic stress conditions, especially persistent perturbations, can make an impact on organismal proteostasis—exacerbating abnormal protein aggregation and causing proteotoxic stress (Hoppe & Cohen, 2020; López‐Otín & Kroemer, 2021). Therefore, stress regulators are likely to help maintain proteostasis and hence promote organismal survival and overall health under proteotoxic stress. In fact it has been established that misfolded and aggregated proteins may arise from oxidative stress and aging but can be prevented by modulation of redox and aging‐related signaling pathways (Wang et al., 2023; Xiao et al., 2022). For example, some antioxidant polysaccharides and peptides are shown to inhibit aggregation of polyglutamine (polyQ), which is involved in several neurodegenerative diseases including Huntington's disease (HD), and thus may have therapeutic potential against neurodegeneration (Ma et al., 2019; Zhang et al., 2012).

In this context, we investigated whether the abalone peptide would have any impact on polyQ‐mediated proteotoxicity. In doing so, we used the transgenic *C. elegans* model AM140 as this strain expresses polyQ expansion tracts with 35 glutamine repeats fused to yellow fluorescent protein (Q35::YFP) in its body wall muscle cells (Morley et al., 2002). When reaching adulthood, AM140 nematodes display a discrete fluorescent aggregate phenotype due to the progressive accumulation of Q35::YFP, a feature that has been used in age‐ and stress‐associated proteotoxicity studies (Haldimann et al., 2011; Hibshman & Goldstein, 2021). Of note, the amount of Q35::YFP aggregates in AM140 nematodes was significantly reduced after treatment with the peptide AbaPep#07 for 5 days as compared to the control (Figure S4). Interestingly, the lifetime survival resilience of AM140 nematodes was ~0.74 relative to the life span of wild‐type or transgenic control nematodes (Table S4), as calculated using the above S/L method (Equation 1).

**FIGURE 2:**
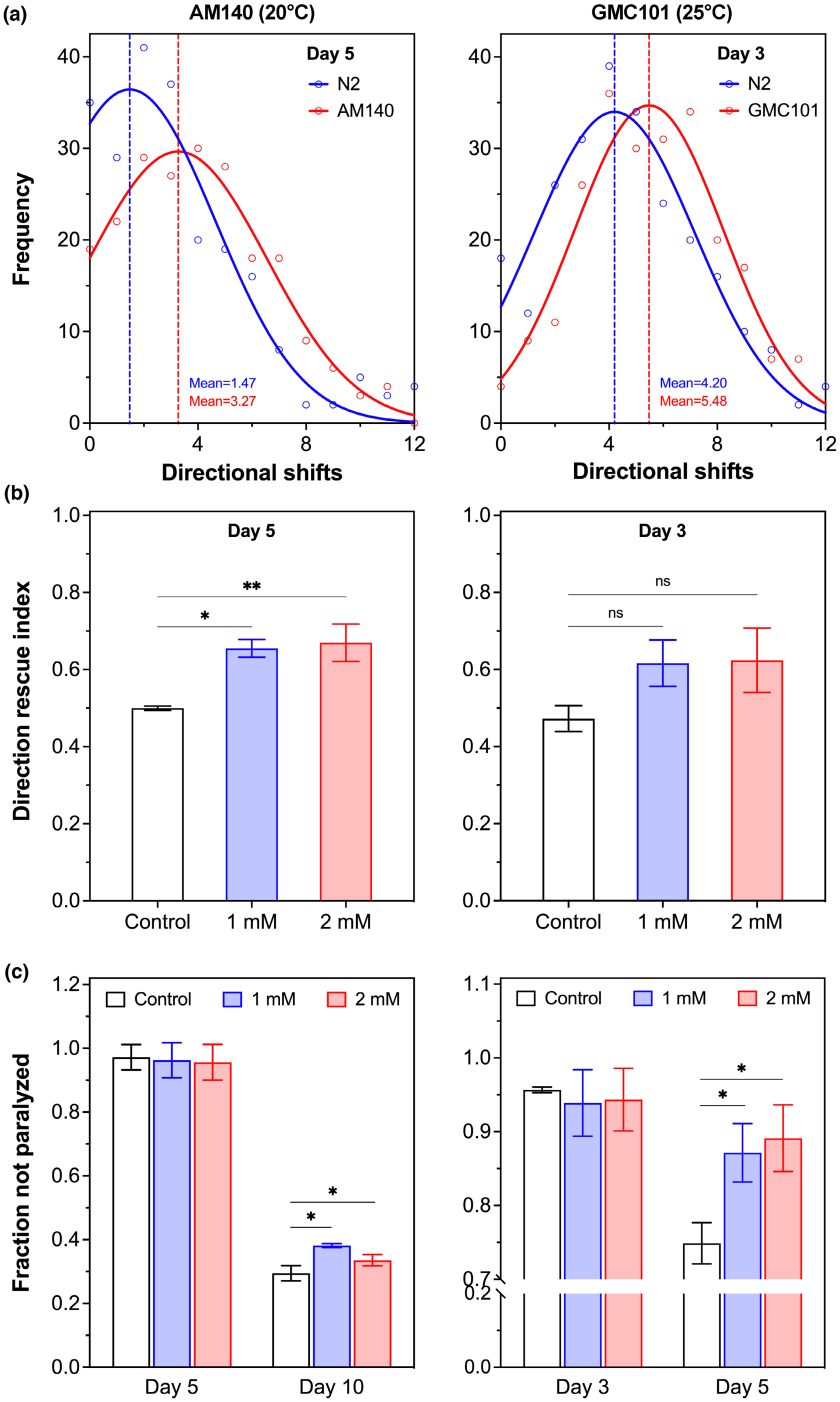
Abalone peptide reduces proteotoxicity‐induced locomotion defects in *C. elegans*. (a) Differential number of directional shifts between wild‐type and proteotoxicity nematodes. Synchronized L4 nematodes were grown at 20°C (N2 and AM140, left panel) or first at 20°C for 12 h and then at 25°C (N2 and GMC101, right panel) for the indicated days. Their movement on agar plates was recorded and analyzed using Movement Tracker to generate average number of directional shifts for wild‐type (N2), polyQ (AM140) and Aβ (GMC101) nematodes, respectively. Data are representative of more than 200 nematodes from five independent experiments for each condition and strain. (b) Rescue of directional defect in *C. elegans* by peptide AbaPep#07. Nematodes were treated with AbaPep#07 and analyzed as in (a). Readouts of directional shifts were compared with the average number of corresponding control (no AbaPep#07) and normalized as direction rescue index (Equation 3) for polyQ (left panel) and Aβ (right panel) nematodes, respectively. Data are mean ± SEM of at least three independent experiments. (c) Increase in fraction of nonparalyzed nematodes by peptide AbaPep#07. Nematodes were treated with AbaPep#07 as above and analyzed with Movement Tracker for paralysis, from which fraction of polyQ (left panel) and Aβ (right panel) nematodes not paralyzed was generated, respectively. Data are mean ± SEM of at least three independent experiments. *p < 0.05; **p < 0.01; ns, not significant.

We then used the Movement Tracker software to analyze the motility of *C. elegans* models, including their shifts of direction, an important indicator of locomotion. As shown in Figure 2a (left panel), the average number of directional shifts of polyQ nematode AM140 on Day 5 of adulthood was much greater than that of the wild‐type nematode N2, consistent with previous reports that the number of directional shifts increases with aging and neurodegeneration (Mouchiroud et al., 2016). Next, to quantify the effect of potential proteostasis regulators on organismal motility deficits in proteotoxically stressed *C. elegans*, we normalized the behavioral data for each experimental group regarding the number of directional shifts. All readouts were compared with the average number of directional shifts in the untreated AM140 nematodes (“control mean”) and thus normalized as a direction rescue index, which was a ratio of the number of *C. elegans* with directional shifts less than the control mean divided by the total number of *C. elegans* in the group (Equation 3). We then analyzed the effect of peptide AbaPep#07 on the behavioral response in the polyQ model AM140 and found that the abalone peptide significantly increased the direction rescue index, with a >1/3 increase (Figure 2b, left panel), demonstrating the potential of peptide‐based stress regulators in the prevention of proteotoxic disorders.

Since the proteotoxicity induced by polyQ aggregation in *C. elegans* will ultimately cause body paralysis (Haldimann et al., 2011; Williams et al., 2020), we further examined whether AbaPep#07 was effective against paralysis using the polyQ model AM140. Interestingly, the fraction of control AM140 nematodes not paralyzed was >0.9 on Day 5 but decreased to ~0.3 on Day 10 (Figure 2c, left panel), suggesting a late‐onset paralysis phenotype in this polyQ35 model as opposed to the relatively early onset of directionality disorder (Figure 2a, left panel) in response to polyQ‐induced proteotoxic stress. This is in line with previous reports showing severe paralysis of AM140 nematodes on Day 10 of adulthood (Williams et al., 2020). As expected, no effect was observed for AbaPep#07 treatment when examined on Day 5. We then analyzed the paralysis rate on Day 10 and found that the fraction of nematodes not paralyzed was significantly increased by AbaPep#07 (Figure 2c, left panel). Collectively, these results demonstrate that the abalone peptide was able to improve the behavioral performance of polyQ‐induced *C. elegans* proteotoxicity model, including direction rescue index and body paralysis rate.

Amyloid‐β (Aβ) peptide plays a critical role in the pathogenesis of Alzheimer's disease (AD), the most common form of dementia. The abnormal accumulation of Aβ is known to impair global protein quality control system, leading to disruption of proteostasis and alteration of stress response. For example, the lifetime survival resilience of *C. elegans* strains expressing Aβ in body wall muscle cells was on average ~ 0.67, which was calculated using the above S/L method and referenced to the life span of wild‐type or transgenic control nematodes (Table S4). The transgenic *C. elegans* strain GMC101 expresses and deposits human Aβ in its body wall muscle cells after temperature upshift (from 20 to 25°C), and shows age‐progressive behavioral phenotypes, including impaired motility and ultimately severe paralysis (Sorrentino et al., 2017). Therefore, we tested whether the abalone peptide was able to improve Aβ‐mediated behavioral deficits using this AD‐like *C. elegans* model. First, we determined the average number of directional shifts of the nematodes on Day 3 after temperature upshift, and found that the average directional shifts of GMC101 nematodes were much more than that of the wild‐type nematodes (Figure 2a, right panel). We further analyzed the effect of AbaPep#07 on the direction rescue index as above and found that the normalized index of GMC101 nematodes treated with the peptide was markedly higher than that of the control GMC101 nematodes, albeit not statistically significant (*p* ≈ 0.2) (Figure 2b, right panel), suggesting the potential of the abalone peptide in preventing Aβ‐induced proteotoxicity.

Previous studies have revealed that *C. elegans* GMC101 starts to become paralyzed from Day 5 post‐temperature upshift due to deposits of Aβ in its body wall muscle cells (Habchi et al., 2017; Sorrentino et al., 2017). Thus, we further tested the potential protective effect of AbaPep#07 on Aβ‐induced paralysis in GMC101 nematodes. As shown in Figure 2c (right panel), only <5% of control GMC101 nematodes were paralyzed on Day 3 after temperature upshift, but ~25% nematodes were paralyzed on Day 5. Unsurprisingly, AbaPep#07 treatment did not increase the fraction of nematodes not paralyzed on Day 3. However, the fraction of nematodes not paralyzed was significantly increased by AbaPep#07 when analyzed on Day 5 after shifting the temperature. Taken together, our results demonstrate that the abalone peptide AbaPep#07 was able to protect *C. elegans* from polyQ‐ and Aβ‐induced behavioral dysfunction, suggesting its potential as a novel proteostasis regulator to increase stress resilience against proteotoxicity.

### 3.3 Abalone peptide increases cost‐free longevity and age‐related physical fitness

**FIGURE 3:**
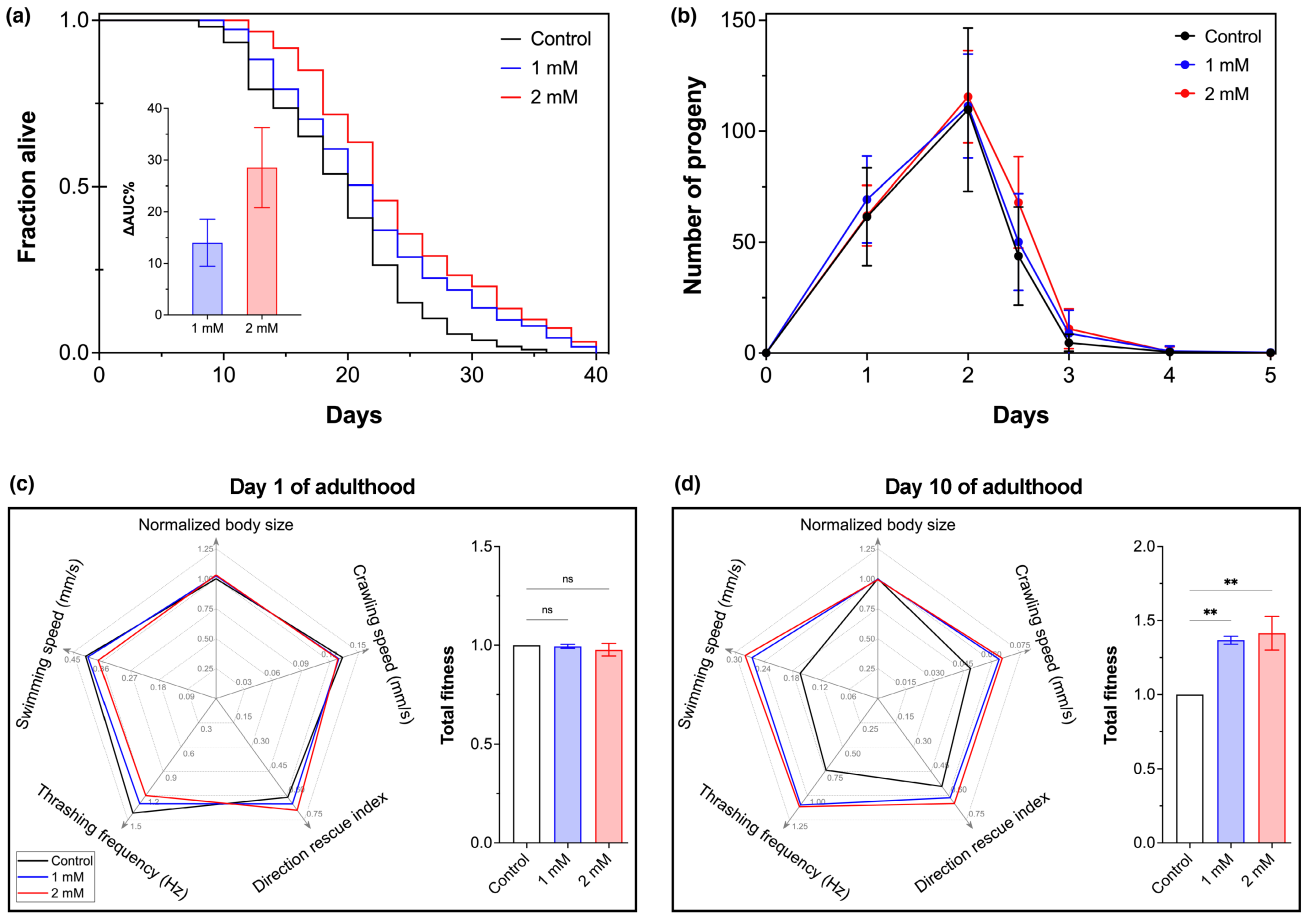
Abalone peptide increases life span and total fitness of aged *C. elegans*. (a) Effect of peptide AbaPep#07 on life span. Wild‐type L4 nematodes were treated with AbaPep#07 at 20°C in 96‐well plates and scored for survival every 2 days. Representative results are presented as Kaplan–Meier survival curves. Inset shows relative life span gain after AbaPep#07 treatment; data are mean ± SEM of three independent experiments. See Table S5 for further information. (b) Effect of peptide AbaPep#07 on brood size. Wild‐type L4 nematodes were treated with AbaPep#07 at 20°C and individually transferred to new NGM plates every day. Progeny was counted 48 h after parent removal. The experiment was performed twice with similar results. (c,d) Effect of peptide AbaPep#07 on total fitness. Wild‐type L4 nematodes were treated with AbaPep#07 until Day 1 (c) or Day 10 (d) of adulthood. To determine body size, crawling speed, and directional shifts, the nematodes were transferred onto solid agar plates for video recording and analysis with Movement Tracker. To determine thrashing frequency and swimming speed, the nematodes were transferred into drops of M9 buffer for video recording and analysis with wrMTrck (ImageJ plugin). The results are presented as a radar chart showing multiple fitness parameters (left panels) and as a single “total fitness” integrating all individual metrics according to Equation 4 (right panels). Data are mean ± SEM of three independent experiments. ns, not significant; **p < 0.01. See Figure S5 and Figure S6 for further information.

As shown above, peptide AbaPep#07 not only increased survival and resilience of wild‐type *C. elegans* under oxidative stress, which is closely associated with aging, but also reduced behavioral dysfunction of proteotoxicity *C. elegans* models expressing polyQ or Aβ, which manifests in an age‐dependent manner. Therefore, we asked whether the peptide could promote longevity under normal conditions. For this, we performed life span assay using wild‐type *C. elegans* and quantitatively estimated the effect of AbaPep#07 on the cumulative life span using relative total survival gain (ΔAUC%), which was defined previously (Guo et al., 2020; Xiao et al., 2022). As expected, the life span of wild‐type nematodes was markedly increased by AbaPep#07 (Figure 3a and Table S5)—the total survival gain was close to 15% and 30% for 1 mM and 2 mM treatments, respectively (Figure 3a inset)—demonstrating the life span‐extending effect of the abalone peptide.

Despite a complex relationship between life span and fertility, decline in reproductive function is one of the earliest hallmarks of aging. According to the evolutionary theory of aging, there is a trade‐off between longevity and reproduction. Indeed, such negative correlation has been observed in *C. elegans* and other animals under various conditions, albeit not always consistent (Lind et al., 2021). Regardless, we tested whether the abalone peptide could affect *C. elegans* fertility, which is an important health indicator. We performed fecundity assays using wild‐type *C. elegans*, and found that neither brood size nor reproductive span of the N2 nematodes was affected by AbaPep#07 (Figure 3b). These results demonstrate that the life span‐extending effect of the abalone peptide was not due to reduced fertility, that is, not at the direct cost of *C. elegans* reproduction.

Another health indicator of utmost importance is body size, which is a fundamental attribute governed by genetic predispositions and environmental factors, including food and nutrition in particular (Wang et al., 2022). Body size is important in the assessment of growth, development, and fitness, particularly at early ages of an organism. However, potential trade‐offs may exist between body size and organismal traits such as developmental timing and life span, for example, negative correlation of body size and longevity (Woodruff et al., 2019). Therefore, we examined whether AbaPep#07 would have any effect on the body size of wild‐type *C. elegans*. We found essentially no difference in body size between the control and peptide‐treated nematodes on either Day 1 or Day 10 of adulthood (Figure 3c,d, respectively), suggesting that the increased life span by the abalone peptide did not result from reduced body size and delayed development.

Progressive decline of physiological functions is a universal feature of aging, which is associated with increasing risk of morbidity and mortality. Therefore, health span rather than mere life span has become a focal point in the combat against aging and associated complications (Campisi et al., 2019; Huang et al., 2022; Longo & Anderson, 2022). A number of health indicators have been used for health span studies in *C. elegans*, including behavioral and physiological phenotypes such as body size, pharyngeal pumping, locomotion, and stress resistance (Wang et al., 2022). For example, the health status of *C. elegans* can be assessed both in liquid media and on solid agar using motor‐related parameters (Figure S5) such as travelled distance, direction of movement, crawling speed, thrashing frequency, body bends, and bend amplitude (Koopman et al., 2020; Laranjeiro et al., 2019). Take, for instance, the thrashing frequency in liquid media (aka body bends per minute), which is recommended as a primary choice to assess health span in *C. elegans* (Koopman et al., 2020). Moreover, the sinusoidal wave‐like crawling movement can provide complementary information about crawling speed on solid agar media. Thus, below we further investigated whether the abalone peptide could improve age‐related physical status of *C. elegans* using the worm tracking systems Movement Tracker (Mouchiroud et al., 2016) and wrMtrck (Nussbaum‐Krammer et al., 2015).

To determine the effect of AbaPep#07 on physical fitness, we compared multiple motility‐related health metrics of wild‐type *C. elegans* treated with or without the peptide. We found almost no difference in all tested locomotory fitness indices between AbaPep#07‐treated and control nematodes on Day 1, including crawling speed, direction rescue index, thrashing frequency, and swimming speed (Figure 3c, left panel; Figure S6). For simplicity, we refer to the entirety of fitness landscape as a single “total fitness,” which is the average of ratios of treated to untreated nematodes for all the above health metrics (Equation 4) and represents the overall health status of *C. elegans*. As expected, the total fitness of *C. elegans* on Day 1 was not changed by the peptide (Figure 3c, right panel). We then further examined the same locomotory parameters on Day 10, and found that the locomotory indicators were significantly improved by AbaPep#07, including swimming speed, crawling speed, and thrashing frequency in particular (Figure 3d, left panel; Figure S6), with the total fitness being increased from 1.0 (i.e., the baseline fitness value of the control) to >1.4—an ~40% increase (Figure 3d, right panel). These data demonstrate that the abalone peptide was capable of promoting overall physical fitness and thus reducing age‐related frailty in *C. elegans*.

In summary, the above results show that the abalone cryptide AbaPep#07 not only promotes survival resilience against oxidative stress but also reduces behavioral dysfunction caused by polyQ‐ and Aβ‐induced proteotoxicity, demonstrating its capacity in stress and proteostasis regulation. Further experiments reveal that the peptide is able to extend the life span of *C. elegans* but does not compromise its growth and reproduction, suggesting its cost‐free prolongevity activity. By targeting locomotion‐associated health span indicators, we also demonstrate that the peptide is capable of increasing physical fitness of *C. elegans*, indicating its potential to reduce age‐related frailty and promote healthy aging. Next, as described below, we sought to identify the molecular mechanisms underlying the protective effects of the abalone peptide against stress and frailty.

### 3.4 Abalone peptide activates the transcription factor SKN‐1 but not DAF‐16

**FIGURE 4:**
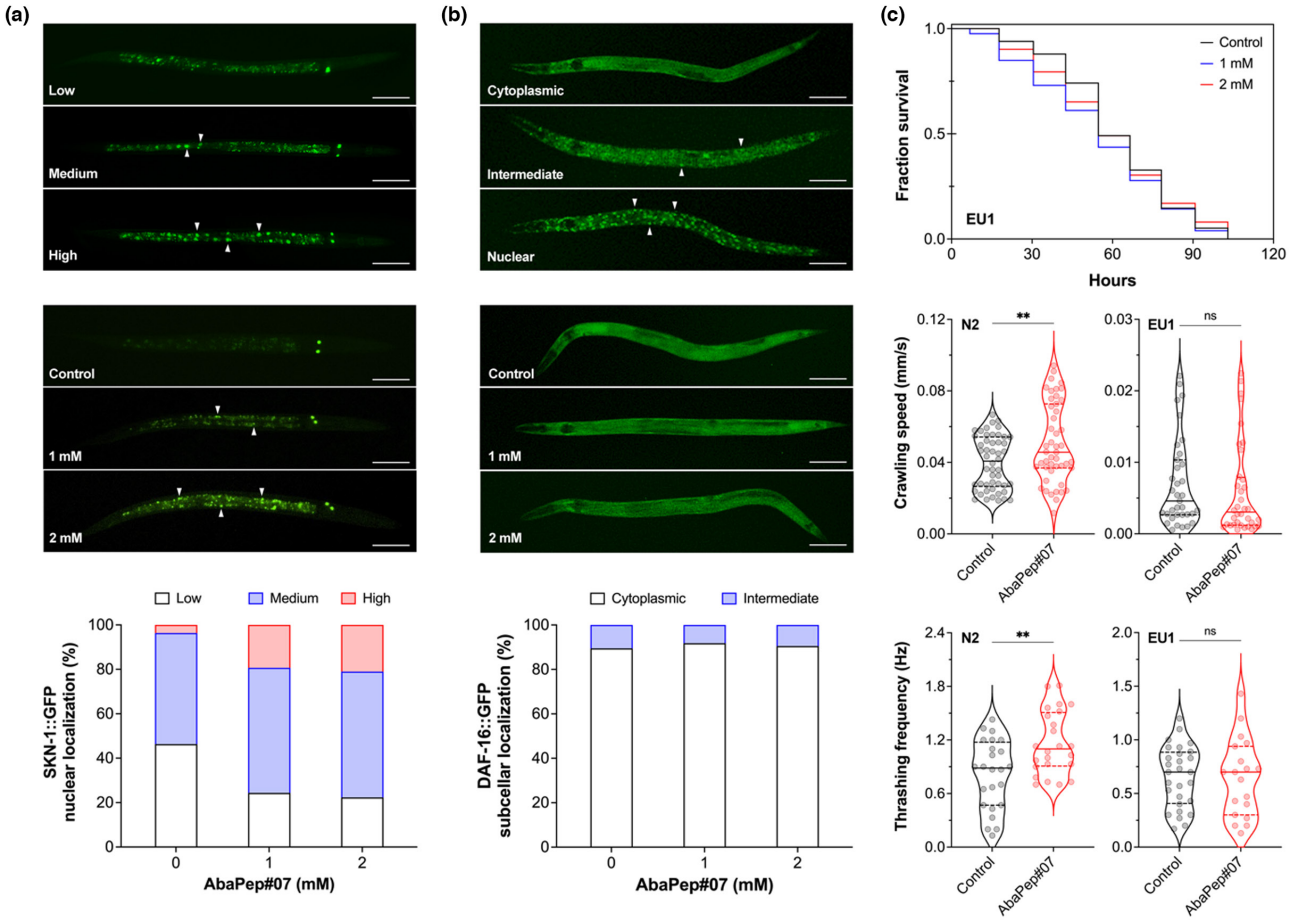
Abalone peptide induces activation of the transcription factor SKN‐1 but not DAF‐16. (a) Effect of peptide AbaPep#07 on nuclear localization of SKN‐1. LG326 nematodes were treated with AbaPep#07 from L1 to L4 and visualized for subcellular localization of SKN‐1::GFP. Top panel: Representative fluorescence micrographs showing “low,” “medium,” and “high” nuclear accumulation of SKN‐1::GFP, respectively. Middle panel: Representative fluorescence micrographs of LG326 nematodes with or without AbaPep#07 treatment. Arrows indicate SKN‐1::GFP aggregated in nuclei. Scale bar, 100 μm. Bottom panel: Percentage of nematodes with “low,” “medium,” and “high” nuclear SKN‐1::GFP accumulation, respectively. Data are from three independent experiments. (b) Effect of peptide AbaPep#07 on nuclear localization of DAF‐16. GR1352 nematodes were treated with AbaPep#07 as in (a) and visualized for subcellular localization of DAF‐16::GFP. Top panel: Representative fluorescence micrographs showing “cytoplasmic,” “intermediate,” and “nuclear” localization of DAF‐16::GFP, respectively. Middle panel: Representative fluorescence micrographs of GR1352 nematodes with or without AbaPep#07 treatment. Arrows indicate DAF‐16::GFP aggregated in nuclei. Scale bar, 100 μm. Bottom panel: Percentage of nematodes with “cytoplasmic,” “intermediate,” and “nuclear” DAF‐16::GFP localization, respectively. Data are from two independent experiments. (c) Effects of peptide AbaPep#07 on oxidative stress survival and physical fitness of *skn‐1* mutant *C. elegans*. Top panel: Representative Kaplan–Meier survival curves for *skn‐1(zu67)* mutant nematodes (strain EU1) treated with or without AbaPep#07 as in Figure 1. See Table S6 for further information. Middle and bottom panels: Violin plots showing physical fitness of wild‐type (N2) and *skn‐1(zu67)* mutant (EU1) nematodes. After treatment with 2 mM AbaPep#07 from L4 to Day 10 of adulthood, crawling speed and thrashing frequency were determined as in Figure 3. Data are representative of three experiments. Solid and dashed lines indicate the median and quartiles, respectively. Statistical significance was analyzed using two‐tailed unpaired *t* test. **p < 0.01; ns, not significant.

Increasing evidence demonstrates that protein hydrolysates and derived peptides are involved in the regulation of signaling pathways associated with stress response and aging process, including nuclear factor erythroid‐2‐related factor (Nrf) and insulin/insulin‐like growth factor signaling (IIS) pathways (Xiao et al., 2022). The transcription factor SKN‐1 (skinhead‐1) is the *C. elegans* ortholog of mammalian Nrf, which is conserved across eukaryotes. SKN‐1/Nrf was originally found to play a pivotal role to maintain redox homeostasis in stress response, but is growingly recognized as a key regulator in many other cellular functions, including proteostasis and life span regulation (Blackwell et al., 2015; Peters et al., 2021). Therefore, to investigate whether SKN‐1 was implicated in the regulatory effect of the abalone peptide on stress resistance and longevity, we used the transgenic *C. elegans* strain LG326, which expresses a SKN‐1::GFP reporter, to visualize nuclear translocation of this transcription factor (An & Blackwell, 2003; Bishop & Guarente, 2007). In response to stimuli, SKN‐1 is translocated from the cytoplasm, where it is in an inactive state, into nucleus and accumulated (Figure 4a, top panel, “low” versus “high”). When the LG326 nematodes were treated with AbaPep#07, an increased relocalization of SKN‐1::GFP fusion proteins to the intestinal cell nucleus was observed (Figure 4a, middle panel)—the percentage of nematodes with high levels of nuclear SKN‐1::GFP was significantly increased as compared to the untreated control (Figure 4a, bottom panel), indicating activation of SKN‐1 by the peptide.

DAF‐16 (abnormal dauer formation‐16) is the *C. elegans* ortholog of mammalian FOXO (forkhead box O) transcription factor, a major IIS downstream component that integrates diverse signaling pathways, including stress response and life span regulation (Xiao et al., 2022; Zhang et al., 2012). To address whether DAF‐16/FOXO was involved in the beneficial effect of the abalone peptide, we used the transgenic *C. elegans* strain GR1352, a model that expresses DAF‐16::GFP reporter, to examine nuclear translocation of the transcription factor (Oh et al., 2005). As shown in Figure 4b (top panel), the GR1352 nematodes in each treatment were classified into three categories, that is, cytoplasmic, intermediate, and nuclear, based on DAF‐16 subcellular localization as previously reported (Oh et al., 2005). The peptide AbaPep#07, however, failed to promote nuclear translocation of DAF‐16::GFP (Figure 4b, middle and bottom panels), despite a significant increase of nuclear localization of the transcription factor being observed in heat‐shocked nematodes (data not shown). Taken together, these results suggest that the stress tolerance and health‐promoting capacities of the abalone peptide are independent of DAF‐16/FOXO but involve SKN‐1/Nrf.

To further investigate the role of SKN‐1/Nrf on the stress resistance and health‐promoting effects of AbaPep#07, we then used *C. elegans* EU1, a loss‐of‐function strain carrying a *skn‐1(zu67)* mutant allele (An & Blackwell, 2003), to examine whether the abalone peptide could increase survival of *skn‐1* mutants under oxidative stress. As shown in Figure 4c (top panel) and Table S6, the survival rate of the *skn‐1* mutant nematodes was not increased by AbaPep#07 when exposed to paraquat, suggesting involvement of SKN‐1 in the above observed antioxidant effect of the peptide (Figure 1c). In addition, treatment with AbaPep#07 was unable to improve physical fitness of aged *skn‐1* mutant *C. elegans* (Day 10 of adulthood), including crawling speed and thrashing frequency, but did increase these health indices in aged wild‐type nematodes (Figure 4c, middle and bottom panels), indicating importance of the transcription factor. Furthermore, in response to stress, PMK‐1, a *C. elegans* homolog of the mammalian mitogen‐activated protein kinase p38, is known to activate SKN‐1 by direct phosphorylation, leading to nuclear translocation and accumulation of the transcription factor (Inoue et al., 2005). Using *C. elegans* KU25, a *pmk‐1(km25)* mutant strain, we found that survival of the mutant nematodes against paraquat‐induced oxidative stress was not increased by AbaPep#07 (Figure S7 and Table S6), further suggesting implication of PMK‐1 and thereby SKN‐1. Collectively, these results demonstrate that the abalone peptide is able to promote stress resistance and physical fitness of *C. elegans* in an SKN‐1‐dependent manner.

### 3.5 Abalone peptide stimulates transcriptional regulation of stress and immune responses

**FIGURE 5:**
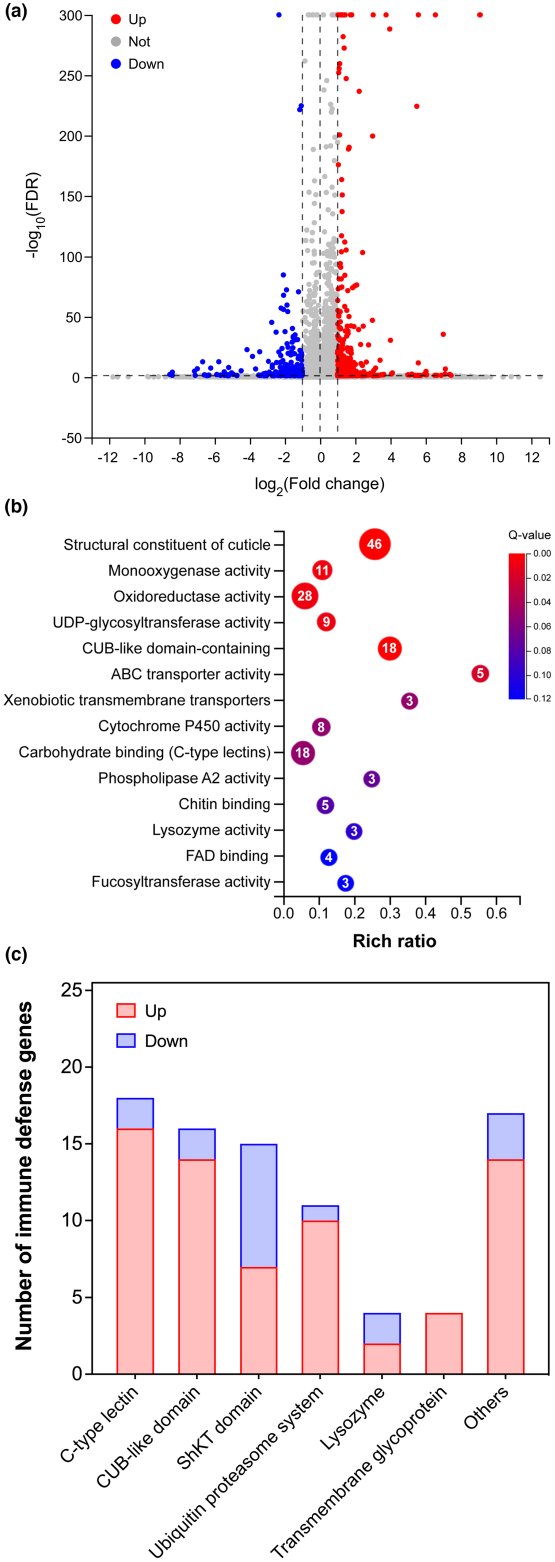
Abalone peptide regulates expression of stress and immune defense genes in *C. elegans*. (a) Volcano plot of genes differentially expressed in control and treated *C. elegans*. Wild‐type L4 nematodes were treated with 2 mM AbaPep#07 until adult Day 10 and used for RNA‐seq analysis. Differential levels of transcripts were defined by FC ≥2 and FDR ≤0.05, which reveals 388 upregulated and 265 downregulated genes (also see Table S7). (b) Top GO terms enriched for biological processes of genes regulated by peptide AbaPep#07. The differentially expressed genes in (a) were used for functional enrichment of GO terms by Phyper with hypergeometric test. The number in a bubble is the number of genes annotated with the corresponding GO term. The bubbles are also color‐coded according to significance; only the terms with *Q*‐value ≤0.05 are considered significant. (c) Innate immune genes identified from the above differentially expressed transcripts. The immune genes regulated by peptide AbaPep#07 are grouped into seven categories based on GO molecular function or InterPro descriptions. Majority of the genes (67/85) are upregulated while the rest are downregulated; many of the genes (26) contain SKN‐1 binding sites (also see Table S7 and Figure S8).

As demonstrated above, the abalone cryptide AbaPep#07 was able to promote organismal health and extend life span of *C. elegans* in a SKN‐1/Nrf‐dependent manner. Therefore, we further asked what other mechanisms were involved in and what genes mediated by SNK‐1/Nrf were associated with the activity of the peptide. To identify genes responsive to the peptide, we performed global transcriptomic analysis using RNA sequencing (RNA‐seq) to compare aged *C. elegans* (Day 10 of adulthood) with and without AbaPep#07 treatment. The genes with fold change (FC) ≥2 and false discovery rate (FDR) ≤0.05 were defined as differentially expressed genes (DEG). By this definition, we found 388 genes were upregulated and 265 genes were downregulated after treatment with the abalone peptide (Figure 5a and Table S7). Gene ontology (GO) enrichment analysis revealed that the top five enriched molecular function terms were “structural constituent of cuticle,” “oxidoreductase activity,” “CUB‐like domain,” “C‐type lectins,” and “monooxygenase activity” (Figure 5b), with a large number of genes that are reported to be involved in immunity, metabolism, and detoxification responses.

It is known that immune system is a key mediator of homeostasis, resilience, and aging, which can be seen in *C. elegans* within a generation and even across generations (López‐Otín & Kroemer, 2021; Willis et al., 2021). Therefore, we first focused on differentially expressed genes that regulate immune response. As shown in Figure 5c, we found that the expression of 85 immune defense genes was regulated by AbaPep#07. Based on InterPro and GO descriptions, these genes were grouped into seven categories, including C‐type lectin, CUB‐like domain, ShKT domain, ubiquitin proteasome system, lysozyme, transmembrane glycoprotein, and other genes. The majority of these genes (67/85) were transcriptionally upregulated. For example, the genes encoding C‐type lectins and CUB‐like domains, which can boost host immune defense against pathogen infections in *C. elegans* (Bolz et al., 2010; Schulenburg et al., 2008), were upregulated. Notably, a number of these immune response genes (26/85) were found to contain SKN‐1 binding sites in their promoters (Figure S8 and Table S7) and thus likely to be its targets (Ewald et al., 2015). These results suggest that the abalone peptide may enhance immune functions via SKN‐1/Nrf, an essential regulator of immune defense response in *C. elegans* and other organisms (Harvey et al., 2011; Papp et al., 2012).

### 3.6 Abalone peptide induces transcriptional activation of metabolic detoxification pathways

**FIGURE 6:**
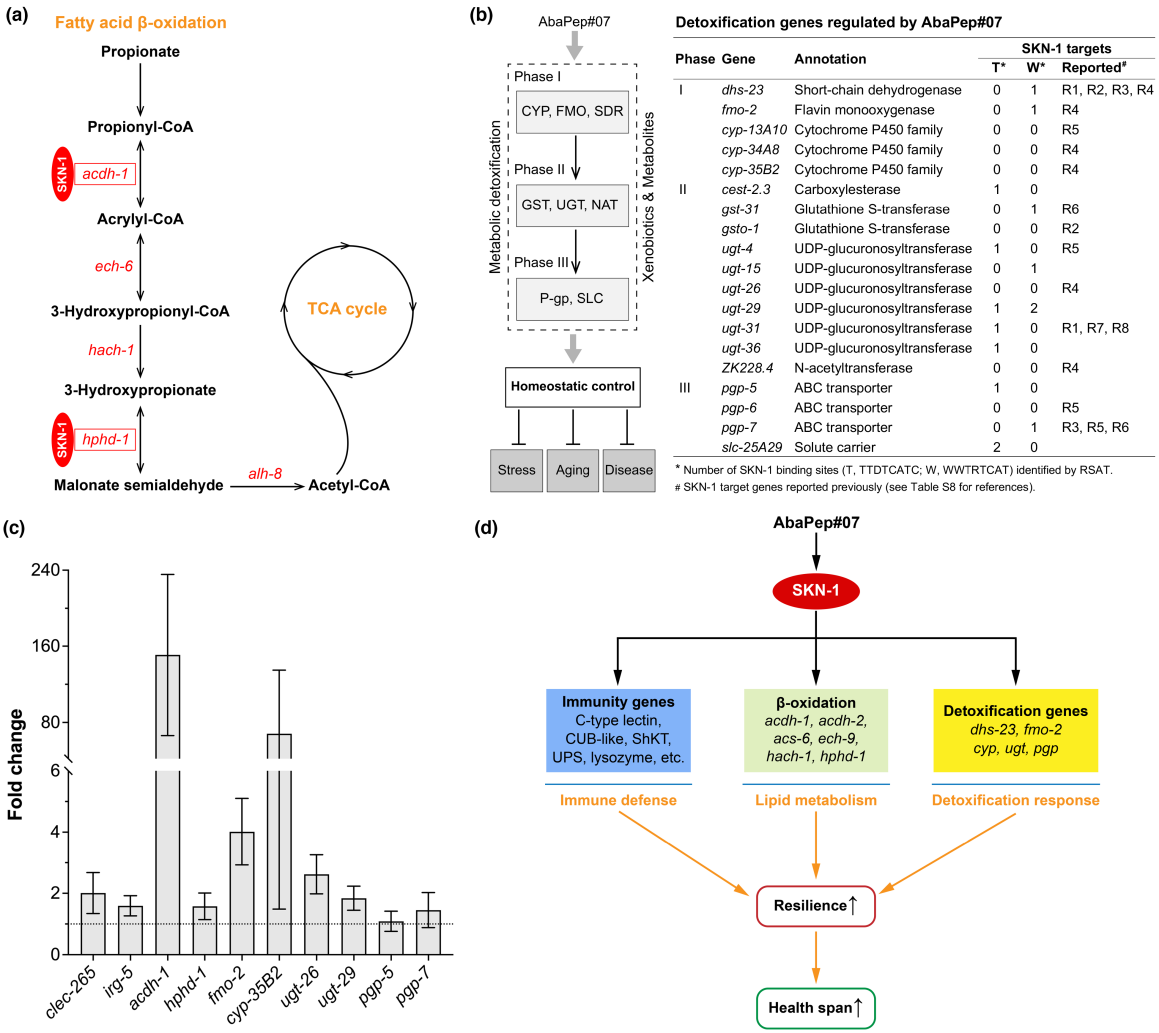
Abalone peptide activates fatty acid metabolism and detoxification genes in *C. elegans*. (a) Schematic of fatty acid metabolism pathways regulated by peptide AbaPep#07. Shown is a summary depiction of mitochondrial β‐oxidation pathway in lipid metabolism involving AbaPep#07‐regulated genes (Table S7). Boxed genes are SKN‐1 targets as predicted by its binding sites (Table S7 and Figure S8). (b) SKN‐1 target and other detoxification genes regulated by peptide AbaPep#07. Left diagram: Overview of phase I, II, and III detoxification enzymes and transporters encoded by genes responsive to AbaPep#07 treatment. Right table: List of AbaPep#07‐regulated genes in the three detoxification phases (also see Table S7). Presence and number of SKN‐1 binding sites for the genes are predicted by RSAT analysis or reported in previous publications (Table S8). (c) Quantitative real‐time PCR analysis of genes selected from transcriptomic data. Wild‐type nematodes were treated as in Figure 5a prior to mRNA preparation and quantitative PCR analysis. Data are presented as mean ± SEM from three independent experiments. (d) Proposed model depicting regulation of SKN‐1‐governed pathways by peptide AbaPep#07. Immune defense, lipid metabolism, and metabolic detoxification are the main pathways identified. Coordinated regulation of these pathways helps to increase stress resilience and health span in *C. elegans*.

Aging and many age‐related diseases are accompanied by progressive loss of metabolic resilience, which can be triggered by intrinsic and extrinsic stress and contributes to organismal frailty (Taylor et al., 2023). Fox example, toxic intermediates, such as the short‐chain fatty acid propionate, may accumulate in lipid metabolism pathway and cause metabolic stress (Zhou et al., 2022). Therefore, maintenance of metabolic homeostasis is of critical importance to health span, whereas *C. elegans* is an emerging model to dissect the underpinning mechanisms of bioactive supplements (Parkhitko et al., 2020; Wang et al., 2022). In this context, we examined the AbaPep#07‐induced genes in *C. elegans* and found that many lipid metabolic genes were regulated by the peptide (Table S7). For example, *acdh‐1* (encoding acyl‐CoA dehydrogenase), *ech‐6* (encoding enoyl‐CoA hydratase 6), *hach‐1* (encoding hydroxyacyl‐CoA hydrolase), and *hphd‐1* (encoding 3‐hydroxypropionate‐oxoacid transhydrogenase), which function in an alternate β‐oxidation‐like propionate breakdown shunt pathway (Nair et al., 2022; Watson et al., 2016), were significantly upregulated, while *alh‐8*—an aldehyde dehydrogenase gene in this pathway—was also slightly upregulated (Figure 6a and Table S7). Likewise, *nhr‐68*, a nuclear hormone receptor gene that is required for propionate shunt activation (Bulcha et al., 2019), was upregulated (Table S7). The expression of other fatty acid β‐oxidation genes, including *acs‐6* (encoding acyl‐CoA synthetase), *acdh‐2* (encoding acyl‐CoA dehydrogenase), and *ech‐9* (encoding enoyl‐CoA hydratase), was also increased (Table S7). Of particular note is the presence of predicted SKN‐1 binding sites in half of the 26 regulated genes, suggesting that these lipid metabolic genes were regulated by the peptide via SKN‐1 signaling in *C. elegans* (Figure 6a and Table S7).

As shown in Figure 4a, the abalone peptide was able to induce the nuclear translocation of SKN‐1, which is a major regulator of detoxification pathways in *C. elegans* (Blackwell et al., 2015; Fukushige et al., 2017). Further to this, the above transcriptomic analysis also indicates that many of the immune defense and lipid metabolism genes regulated by the peptide are predicted to be the targets of SKN‐1 (Figure 5, Figure S8 and Table S7). Importantly, activation of metabolic response may help reduce intracellular accumulation of toxic intermediates such as propionate and 3‐hydroxypropionate (Nair et al., 2022; Zhou et al., 2022). Thus, we asked whether and what detoxification genes were regulated by the peptide. Previous studies have demonstrated that SKN‐1/Nrf directly induces expression of detoxification enzymes, including short‐chain dehydrogenases/reductases (SDR) and cytochrome P450 (CYP) enzymes in phase I, glutathione‐S‐transferases (GST) and UDP‐glucuronosyltransferases (UGT) in phase II, and ATP‐binding cassette (ABC) transporters in phase III, in response to endogenous and exogenous stress (Blackwell et al., 2015; Fukushige et al., 2017; Shore et al., 2012). Here, our transcriptomic profiling revealed that more than 40 of the differentially expressed genes are those involved in the above three‐phase detoxification system, and the majority are SKN‐1/Nrf‐dependent detoxification genes, which contain SKN‐1 binding sites in their promoters as predicted by RSAT (Turatsinze et al., 2008) or are reported to be SKN‐1 targets in previous publications (Figure 6b, Table S7 and Table S8). Collectively, it is likely that AbaPep#07 activates SKN‐1, which in turn mounts a detoxification response, leading to stress resistance in *C. elegans*.

To further verify the genes regulated by the abalone peptide, we performed quantitative real‐time PCR to examine the differentially expressed genes revealed by the above global transcriptomic analysis, particularly those predicted to be the target genes of the transcription factor SKN‐1/Nrf. As shown in Figure 6c, the transcript levels of several tested genes were increased (>1.5‐fold change) by AbaPep#07, including genes implicated in immune defense (*clec‐265* and *irg‐5*), fatty acid β‐oxidation (*acdh‐1* and *hphd‐1*), and metabolic detoxification (*fmo‐2*, *cyp‐35B2*, *ugt‐26*, *ugt‐29* and *pgp‐7*), consistent with the RNA‐seq transcriptomic data. Since the activation of SKN‐1 has been reported to induce metabolic stress response that drives depletion of somatic lipids and impairs health in *C. elegans* (Nhan et al., 2019), we asked whether AbaPep#07 would have an effect on lipid stores in the nematodes. For this, we performed fatty acid profiling of *C. elegans* by gas chromatography–mass spectrometry (GC–MS) and found that the fraction of a given fatty acid relative to the total fatty acid pool was not significantly changed by the peptide (Figure S9). This is in agreement with the RNA‐seq data, which showed little effect on fat synthesis genes by the peptide—while the Δ9‐fatty acid desaturase *fat‐7* is downregulated, other lipogenesis associated genes are not affected or only slightly regulated by AbaPep#07 (Table S7 and Figure S9).

In summary, a variety of genes associated with immune defense, lipid metabolism, and metabolic detoxification are regulated by the abalone peptide AbaPep#07. Of note, a detailed analysis on the regulated genes points to the transcription factor SKN‐1/Nrf as indicated by motif similarities of binding sites or information from previous studies. Importantly, the peptide is also shown to promote nuclear localization and thus activation of SKN‐1/Nrf, a master regulator of stress response and damage control. Taken together, our results suggest that the novel abalone cryptide is able to reboot defense, metabolic and detoxification genes largely through the transcriptional governance of SKN‐1/Nrf and promote organismal resilience and physical health in aged *C. elegans* (Figure 6d).

## 4 DISCUSSION

Aging is accompanied by declining resilience and increasing frailty of an organism, thereby weakening its response to deleterious damages caused by exogenous and endogenous stressors (López‐Otín & Kroemer, 2021; Taylor et al., 2023). To maintain homeostatic resilience, an organism must constantly sense, integrate and respond to stress signals as various stresses persist throughout its entire life (Costa‐Mattioli & Walter, 2020; Huang & Tunnacliffe, 2006; Kaczmarek et al., 2019). The organism is likely to uphold its homeostatic integrity when challenged by a tolerable stress, but may establish a new homeostasis in response to a persistent severe stress by, for example, recalibrating metabolic balance. Failure of such resilience—the ability to minimize or avoid deleterious stress effect—the organism may lose physiological integrity, leading to functional impairment, severe frailty, and eventual death (Huang et al., 2010; Huang & Tunnacliffe, 2006; Peters et al., 2021). Notably, quantitative modeling of resilience (e.g., “resilience index”) is growingly being recognized as a priority for prediction and measurement of stress resistance and for identification of resilience factors and mechanisms (Hampel et al., 2019; Taylor et al., 2022). For example, transcriptional resilience parameters are used to predict thermotolerance in *C. elegans* (Jovic et al., 2019). However, since the desired endpoint of stress response is survival of organisms, quantitative framework to couple organismal resilience to survival phenotype is of paramount interest.

In this study, we use a conceptual loss‐or‐gain model in *C. elegans* to quantify organismal survival resilience based on its stress resistance capacity, that is, the cumulative survival of stressed population relative to normal population across their entire life (S/L ratio) (Figure S1). In this scenario, an S/L ratio of <1 or >1 indicates a loss or gain, respectively, of the overall survival resilience. This approach avoids the need to quantify individual variables by de facto integration of sometimes evasive resilience properties, including stress tolerance, physiological adaptation, and homeostasis restoration, which are the three main resilience hallmarks (Taylor et al., 2022). When exposed to an increasing dose of paraquat, the survival resilience of *C. elegans* gradually but consistently declines with the increasing oxidative stress triggered by paraquat insult (Figure 1a and Table S1). Interestingly, the survival resilience is also compromised by proteotoxic stress caused by polyQ and Aβ as shown by reduced life span of transgenic AM140 and GMC101 strains, respectively, relative to that of wild‐type *C. elegans* (Table S4). These results clearly establish the feasibility of using this simple loss‐or‐gain model to reflect organismal survival resilience against both external and internal stresses.

Oxidative stress resistance is generally regarded as a likely, albeit not necessarily accurate, predictor of antiaging capability, while life span extension is considered an unequivocal antiaging index (Wang et al., 2014; Xiao et al., 2022). Using the above loss‐or‐gain model, we show that the abalone cryptide AbaPep#07 significantly increases the survival resilience of wild‐type *C. elegans* against oxidative stress (Figure 1c). As expected, AbaPep#07 can increase the life span (up to 30% ΔAUC%) (Figure 3a), boost age‐related physical fitness (Figure 3d), and also rescue HD‐ and AD‐like behavioral deficits of *C. elegans* (Figure 2), demonstrating its antiaging capacity. On the other hand, virtually all hallmarks of aging and decline of age‐related fitness are connected to, or in fact intertwined with, progressive metabolic dysregulation and dysfunction (Finkel, 2015; López‐Otín et al., 2016). For example, altered levels of particular metabolic intermediates during aging are likely to cause intracellular stress and result in toxic outcomes. In this regard, AbaPep#07 is found to increase the expression of genes involved in fatty acid β‐oxidation (e.g., propionate breakdown pathway) (Figure 6 and Table S7), suggesting its potential to recalibrate metabolic integrity and ultimately promote overall health.

The short‐chain fatty acid propionate is a metabolic intermediate of branched‐chain amino acids, odd‐chain fatty acids, and cholesterol. Its excessive accumulation is known to disrupt mitochondrial homeostasis, causing stress toxicity (Na et al., 2020; Revtovich et al., 2019). Interestingly, persistent high level of propionate is found to activate a β‐oxidation‐like propionate breakdown shunt pathway—comprising *acdh‐1*, *ech‐6*, *hach‐1*, *hphd‐1,* and *alh‐8*—in *C. elegans* fed with the standard, but vitamin B12‐deficient, diet *E. coli* OP50 (Na et al., 2020; Nair et al., 2022; Watson et al., 2016). In this study, we find that these genes, together with *nhr‐68*—a gene encoding a nuclear hormone receptor that is required for the propionate shunt activation (Bulcha et al., 2019)—are upregulated by AbaPep#07 (Figure 6a and Table S7). Also upregulated are the genes coding for other fatty acid β‐oxidation enzymes, including *acs‐6*, *acdh‐2,* and *ech‐9* (Table S7). These results demonstrate activation of the fatty acid β‐oxidation pathway by AbaPep#07 and warrant future investigations at metabolic level. Intriguingly, dietary restriction of branched‐chain amino acids is recently reported to have beneficial effects on frailty and life span in mice (Richardson et al., 2021). Consistent with this, AbaPep#07 is shown to promote physical fitness, alleviate neurodegeneration–like symptoms, and extend life span of *C. elegans* (Figure 2 and Figure 3). Together, these findings warrant further investigation to develop AbaPep#07 as potential antiaging and antineurodegenerative therapeutics and to explore the fatty acid β‐oxidation pathway for discovery of interventions against aging‐related disorders.

In addition to lipid metabolism, other metabolic pathways are also enriched by AbaPep#07 (Table S7). For example, >40 enrichments are genes encoding phase I, II, and III detoxification enzymes (Blackwell et al., 2015; Fu et al., 2020; Fukushige et al., 2017). In phase I, the best known enzymes are CYP, which correlate positively with stress resistance and life span in both invertebrates and mammals (Fu et al., 2020; Steinbaugh et al., 2012). In phase II, the multifunctional GST enzymes respond to toxic chemicals and oxidative stress (Ferguson & Bridge, 2019; Fukushige et al., 2017; Shore et al., 2012), while the conserved UGT enzymes target toxic metabolites and xenobiotics, with some UGT isoforms acting in coordination with CYP and GST (Blackwell et al., 2015; Shore et al., 2012). In phase III detoxification, the ABC transporters, for example, the multidrug resistance efflux pump P‐glycoprotein (P‐gp), play a central role to eradicate xenobiotics (Ai et al., 2013; Voss et al., 2020). Of note, several genes in each detoxification phase are differentially regulated by AbaPep#07, including those encoding CYP, GST, UGT, and P‐gp (Figure 6), demonstrating their involvement in AbaPep#07 bioactivities. Adding further intrigue to the metabolic detoxification, mutations in *glct‐3*, a gene encoding a protein of the glucuronosyltransferase family that includes UGT enzymes, are recently shown to modify the sensitivity of *C. elegans* to propionate despite no direct association between UGT and propionate breakdown pathway (Na et al., 2020). Taken together, these findings suggest that a more robust metabolic control system is likely involved in the integration of aging and stress signals but can be manipulated by interventions such as the abalone peptide.

Increasing evidence support the implication of SKN‐1/Nrf in metabolic surveillance, including responses to excessive metabolites, disease proteins, aging stress, and xenobiotic insults, despite being originally discovered as an antioxidant transcription factor (Blackwell et al., 2015; Dodson et al., 2019; Frankino et al., 2022; Peters et al., 2021). In this study, a large number of AbaPep#07‐regulated genes are the reported targets or contain predicted binding sites of SKN‐1/Nrf, including many of the differentially expressed genes in immune defense (26 out of 85) and lipid metabolism (13 out of 26) as well as the majority of the 40 regulated genes in the three phases of metabolic detoxification (Figure 5, Figure 6, and Table S7). Equally important, AbaPep#07 is shown to activate SKN‐1 as indicated by the increased nuclear translocation of SKN‐1::GFP fusion proteins (Figure 4a), while loss‐of‐function *skn‐1* mutation is found to abolish the beneficial effects of AbaPep#07 against oxidative stress and age‐related locomotory fitness as demonstrated in the *skn‐1(zu67)* mutant EU1 (Figure 4c). On the other hand, a recent study has used a low dose of paraquat (75 μM) and a gain‐of‐function mutant [*skn‐1(lax188)*] to induce activation of SKN‐1 and performed RNA‐seq analysis (Nhan et al., 2019). In comparison, 21 AbaPep#07‐regulated genes are found also differentially expressed in the paraquat‐exposed wild‐type nematodes (versus non‐exposed control) and in the *skn‐1(lax188)* mutants (versus wild‐type nematodes). Intriguingly, the majority of these genes (18 out of 21) are in an opposite regulation pattern between AbaPep#07‐treated and paraquat‐exposed nematodes, while about half of these genes (11 out of 21) are regulated in the same direction between AbaPep#07‐treated and *skn‐1(lax188)* mutant nematodes, further supporting the regulation of SKN‐1‐associated genes by AbaPep#07. Together, these findings demonstrate that AbaPep#07 is able to promote stress resistance and physical fitness via SKN‐1/Nrf‐governed regulation of gene expression involving immune defense, lipid metabolism, and metabolic detoxification.

The sequence of AbaPep#07 matches hemocyanins, the oxygen carrier proteins found in arthropod and mollusk hemolymph (Gianazza et al., 2021). Surprisingly, the antioxidant potential of hemocyanins or hemocyanin‐derived peptides is uninvestigated. The present study is, to our knowledge, the first report on the antioxidant activity of peptides derived from hemocyanins. Nevertheless, hemocyanins are known to enhance immune system function against pathogenic attack and environmental stimuli in arthropods and mollusks (Coates & Nairn, 2014; Gianazza et al., 2021). Notably, several peptides derived from abalone and other hemocyanins have shown antimicrobial properties (Zhuang et al., 2015). In a broader context, antimicrobial host peptides isolated from various species, as well as those released from precursor proteins of mammals (including humans) during inflammation, have been found able to combat infections and thus prompted intense development for clinical applications (Mookherjee et al., 2020). Interestingly, plants can also release immunomodulatory peptides by activating proteases upon physical damage and elicit an innate immune‐like defense response (Hander et al., 2019). In this study, AbaPep#07 is shown to regulate 85 immune defense genes (Figure 5c and Table S7), which are reported to play significant roles in *C. elegans* innate immune response (Bolz et al., 2010; Schulenburg et al., 2008). Together, these findings support that peptide fragments encrypted in native proteins can be released and exert bioactive functions, for example, immune defense and homeostatic resilience by AbaPep#07. However, how to crack cryptomes and identify desired peptides from large numbers of such peptides (e.g., Guo et al., 2020; Zhang et al., 2021) remains to be explored.

To cope with a persistent stress, an organism may adopt two distinct but also overlapping metabolic strategies—“caretaker” and “gatekeeper” mechanisms. In response to most stresses, a caretaker regulatory network—the general, cross‐stress responses that maintain overall homeostasis—is initiated (Huang et al., 2010; Huang & Tunnacliffe, 2004; Wu et al., 2020). On the other hand, in a specified context triggered by a persistent and severe stressor, an elaborate gatekeeper resistance program is invoked to control stress damages. Paradoxically, the context‐specific gatekeeper response that is not universally shared by different stresses may prompt a robust, albeit not necessarily optimum, metabolic state in the organism and act like a caregiver to resist other stresses as well. In this regard, anhydrobiosis offers a supreme example. Anhydrobiotic quiescence enables organisms to withstand a variety of prolonged harsh stress conditions—not only extended desiccation per se but also other chemical and physical extremes (Boothby et al., 2017; Huang & Tunnacliffe, 2006; Kaczmarek et al., 2019). Such seemingly passive (e.g., extremely low metabolism) but successful defense strategies mounted by anhydrobiotes, including the underlying molecules (e.g., glycans and peptides), may be used via anhydrobiotic engineering to help instigate proactive combat against stresses in non‐anhydrobiotes (Boothby et al., 2017; Hibshman & Goldstein, 2021; Huang et al., 2010). In this study, AbaPep#07 not only boosts the caretaker mechanisms against oxidative stress but also functions as a gatekeeper inducer to protect against proteostasis disruption. Considering the extensive transcriptional reprogramming observed (Table S7), AbaPep#07 may also have beneficial implications in a broader stress context. Nevertheless, whether it works in more extreme conditions, for example, anhydrobiotic stress, and how to translate such mechanisms into biomedical applications need further investigation.

In conclusion, we have successfully used loss or gain of stress survival, represented by the ratio of cumulative stress span to life span, to quantify organismal resilience. As a proof of concept, this is demonstrated in *C. elegans* exposed to increasing oxidative stress induced by exogenous paraquat and with proteotoxic stress caused by endogenous polyQ and Aβ aggregation, respectively. Based on this, we reveal that the abalone peptide AbaPep#07 not only promotes survival resilience against paraquat‐induced oxidative stress but also confers protection against polyQ‐ or Aβ‐mediated behavioral dysfunction in *C. elegans*, indicating its capacity against stress and neurodegeneration. Importantly, we further show that the peptide increases physical fitness of aged *C. elegans* and extends life span without compromise of growth and reproduction, demonstrating its cost‐free prolongevity activity. Moreover, we reveal that the peptide induces differential expression of a number of genes, including innate immune, lipid metabolism, and metabolic detoxification genes, which are associated with the SKN‐1/Nrf transcription factor. Collectively, our findings demonstrate that the novel abalone cryptide AbaPep#07 increases stress survival resilience and cost‐free longevity via SKN‐1/Nrf‐governed transcriptional reprogramming and provide an insight into the neuroprotective and health‐promoting potential of cryptides as proteostasis regulators.

## AUTHOR CONTRIBUTIONS

Conceptualization: Zebo Huang, Qiangqiang Wang, and Liangyi Wang; methodology: Qiangqiang Wang, Liangyi Wang, Ziliang Huang, Kunping Li, and Ming Liang; investigation and data curation: Qiangqiang Wang, Liangyi Wang, Ziliang Huang, Huihui Liu, Yue Xiao, Yi Yu, Ning Luo, and Ajay Mishra; formal analysis: Qiangqiang Wang, Liangyi Wang, and Yi Yu; writing—original draft: Qiangqiang Wang and Liangyi Wang; writing—review & editing: Zebo Huang, Qiangqiang Wang and Ajay Mishra; supervision: Zebo Huang and Ming Liang. All authors approved the final draft for publication.

## CONFLICT OF INTEREST STATEMENT

The authors declare no competing interests.

## Supporting information


Data S1.

Table S7.

## Data Availability

RNAseq data are deposited in the Gene Expression Omnibus (GEO) repository with accession No. GSE246759. The data supporting the findings of this study are available from the corresponding author upon reasonable request.
