# Supplementary material for: Abalone peptide increases stress resilience and cost‐free longevity via SKN‐1‐governed transcriptional metabolic reprogramming in C. elegans
Source: Aging Cell. 2023 Nov 22;23(2):e14046. doi: 10.1111/acel.14046 (PMC10861207; doi:10.1111/acel.14046)
Supplement: Supplementary file 1 — Data S1. [file ACEL-23-e14046-s002.pdf]

Supporting Information for

Abalone peptide increases stress resilience and cost-free longevity via SKN-1-governed transcriptional metabolic reprogramming in *C. elegans*

Qiangqiang Wang[1,2] | Liangyi Wang[1,3] | Ziliang Huang[1,3] | Yue Xiao[1,2] | Mao Liu[1,2] | Huihui Liu[1,3] | Yi Yu[4] | Ming Liang[4] | Ning Luo[5] | Kunping Li[5] | Ajay Mishra[6] | Zebo Huang[1,2,3]

[1]Institute for Food Nutrition and Human Health, School of Food Science and Engineering, South China University of Technology, Guangzhou, China

[2]Guangdong Province Key Laboratory for Biocosmetics, Guangzhou, China

[3]Center for Bioresources and Drug Discovery, School of Biosciences and Biopharmaceutics, Guangdong Pharmaceutical University, Guangzhou, China

[4]Research and Development Center, Infinitus (China) Company Ltd., Guangzhou, China

[5]Institute of Chinese Medicinal Sciences, Guangdong Pharmaceutical University, Guangzhou, China

[6]European Bioinformatics Institute, Cambridge, UK

Correspondence

Zebo Huang, School of Food Science and Engineering, South China University of Technology, Guangzhou 510641, China.

Email: huangzebo@scut.edu.cn

Qiangqiang Wang and Liangyi Wang contributed equally to this work.

List of supplementary data

FIGURE S1 A loss-or-gain model for quantification of stress survival resilience.

FIGURE S2 Survival resilience of *C. elegans* treated with 0–1 mM paraquat.

FIGURE S3 Effect of selected peptides on *C. elegans* survival against oxidative stress induced by paraquat.

FIGURE S4 Effect of abalone peptide on polyQ aggregation in *C. elegans*.

FIGURE S5 Measurement of motility-related health metrics of *C. elegans* using worm tracking systems.

FIGURE S6 Effect of abalone peptide on fitness metrics of *C. elegans*.

FIGURE S7 Effect of abalone peptide on survival of *pmk-1* mutant *C. elegans* against oxidative stress.

FIGURE S8 Consensus SKN-1-binding motifs.

FIGURE S9 Effect of abalone peptide on expression of lipogenesis genes and composition of fatty acids in *C. elegans*.

TABLE S1 Survival resilience of *C. elegans* to exogenous oxidative stress induced by paraquat.

TABLE S2 Statistics for *C. elegans* survival assays under paraquat-induced oxidative stress.

TABLE S3 Peptides identified in abalone protein hydrolysate and tested for antioxidant capacity.

TABLE S4 Survival resilience of *C. elegans* to endogenous proteotoxic stress.

TABLE S5 Statistics for *C. elegans* life span assays.

TABLE S6 Statistics for survival assays of mutant *C. elegans* under paraquat-induced oxidative stress.

TABLE S7 List of differentially expressed genes (separate Excel file).

TABLE S8 References for SKN-1-targeted genes.

TABLE S9 List of primers used for quantitative real-time PCR analysis.

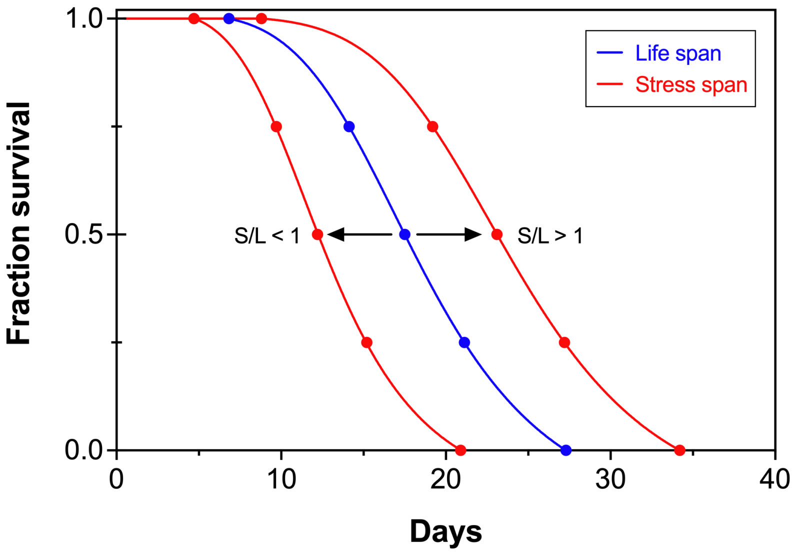

FIGURE S1 A loss-or-gain model for quantification of stress survival resilience. The area under the curve (AUC) is used to quantify the cumulative survival capacity of *C. elegans* against stress. The survival resilience (Equation 1) is defined as AUC ratio of stress span to life span (S/L), where S/L<1 or >1 indicates a resilience loss or gain, respectively.

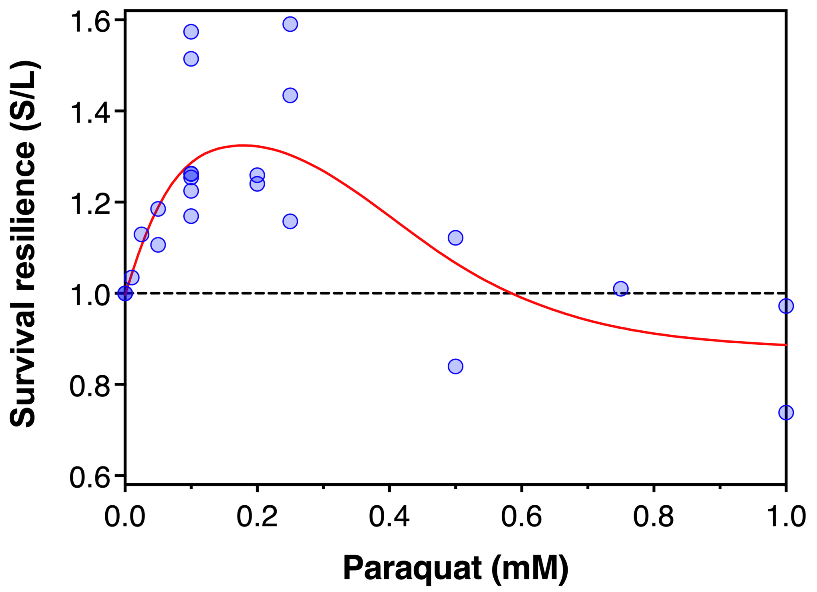

FIGURE S2 Survival resilience of *C. elegans* treated with 0–1 mM paraquat. See Table S1 and also Figure 1a.

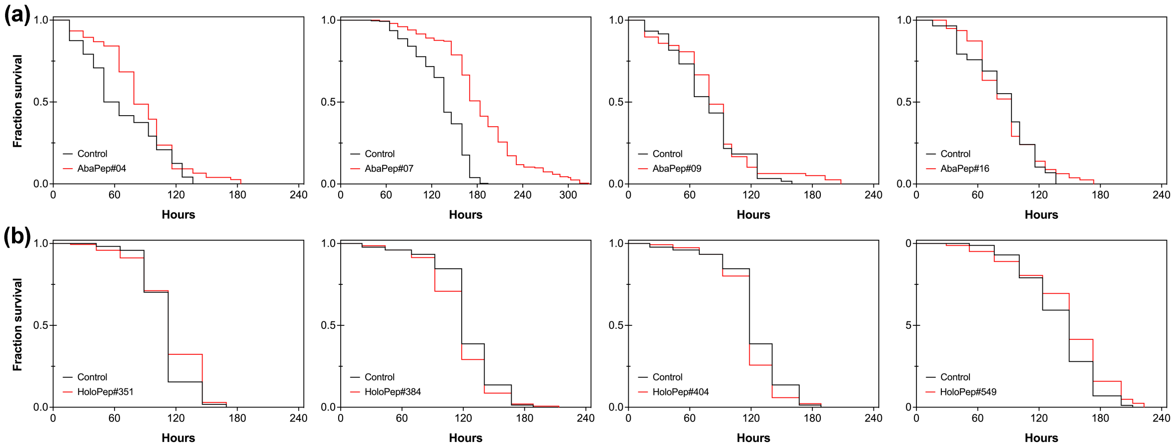

(c) Information of screened peptides

Peptide ID	Source of origin	Sequence	Number of amino acids	MW (Da)	Branched-chain amino acids (%)	Aromatic amino acids (%)	GRAVY*,#	Theoretical pI*
AbaPep#04	Abalone	IGVNIPWH	8	935.1	37.5	12.5	0.400	6.74
AbaPep#07	Abalone	SETYELRK	8	1025.1	12.5	12.5	-1.800	5.86
AbaPep#09	Abalone	KDLELAVISH	10	1124.3	40.0	0.0	0.320	5.32
AbaPep#16	Abalone	VLAYEPVWAIGTGK	14	1503.8	28.6	14.3	0.543	5.97
HoloPep#351	Sea cucumber	DLTDYLMK	8	998.2	25.0	12.5	-0.425	4.21
HoloPep#384	Sea cucumber	ILHRVPTM	8	966.2	37.5	0.0	0.550	9.76
HoloPep#404	Sea cucumber	NMQTHPTYL	9	1104.3	11.1	11.1	-0.978	6.74
HoloPep#549	Sea cucumber	YGNEGALR	8	878.9	12.5	12.5	-1.000	6.00

* These data were obtained by using the ProtParam tool (https://web.expasy.org/protparam). # A grand average of hydropathicity (GRAVY) value above 0 indicates hydrophobicity while that below 0 implies hydrophilicity; an increasing positive score suggests a greater hydrophobicity.

FIGURE S3 Effect of selected peptides on *C. elegans* survival against oxidative stress induced by paraquat. Wild-type L4 nematodes were pretreated with selected abalone (a) and sea cucumber (b) peptides for 24 h and then exposed to 50 mM paraquat. Live and dead nematodes were scored every 12 h or 24 h until all dead. Representative results are presented as Kaplan-Meier survival curves. Information of the tested peptides are shown in (c).

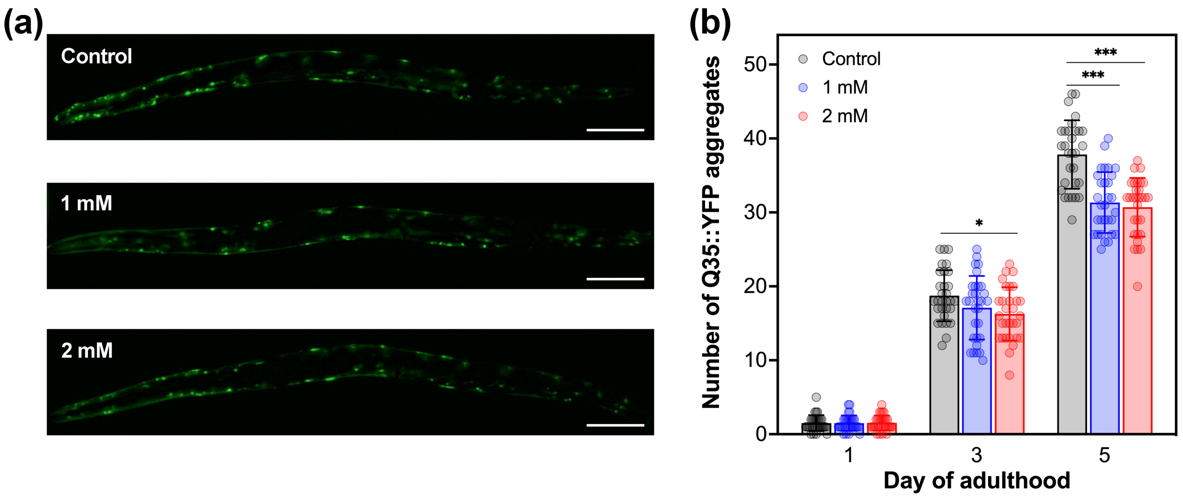

FIGURE S4 Effect of abalone peptide on polyQ aggregation in *C. elegans*. (a) Representative fluorescence micrographs of AM140 nematodes on day 5 of adulthood showing Q35::YFP aggregates in body wall muscle cells. Scale bar, 100 μm. (b) Quantification of Q35::YFP aggregates in body wall muscle cells of AM140 nematodes treated with or without AbaPep#07 at 20°C from L4 stage. Data are representative of three independent experiments and presented as mean ± SD (n ≥ 30 nematodes for each treatment). * p<0.05; *** p<0.001.

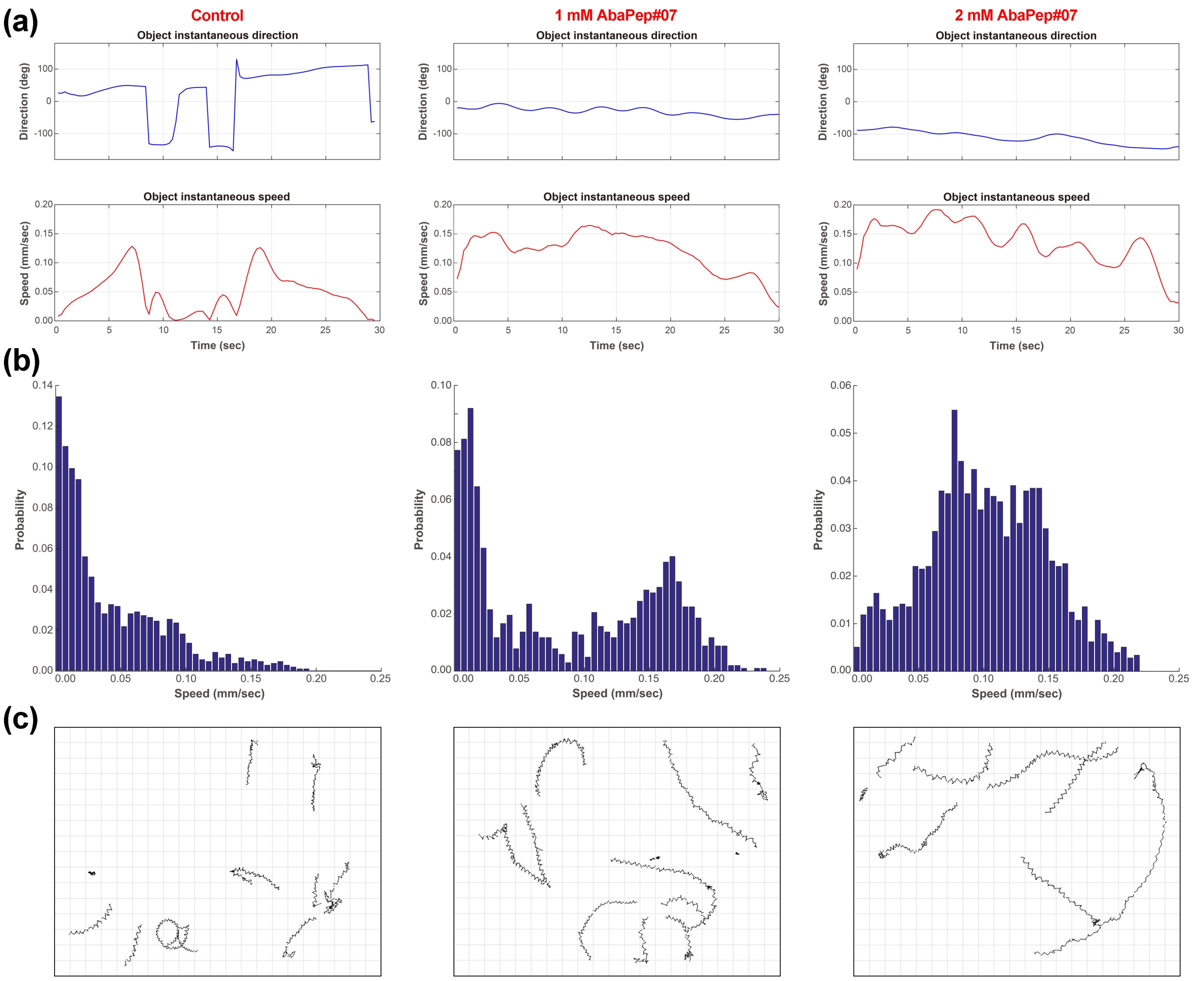

FIGURE S5 Measurement of motility-related health metrics of *C. elegans* using worm tracking systems. (a) Instantaneous direction (upper panels) and instantaneous speed (lower panels) of individual nematode on solid agar plate are measured by Movement Tracker and plotted against time for the duration of tracking. For instantaneous direction, each time the line meets the *x* axis it is recorded as a shift in direction of the nematode. (b) Distribution of crawling speed is measured on solid agar plate by Movement Tracker. Of note, the speed distribution of nematodes treated with peptide AbaPep#07 markedly shifts toward the right side (larger value) as compared to the control. (c) Shown are representative images of nematode swimming tracks analyzed in a drop of M9 buffer by wrMtrck. In the above experiments, wild-type L4 nematodes with or without AbaPep#07 treatment were grown at 20°C until day 10 of adulthood. The nematodes were then collected and transferred to a food-free NGM agar plate or a drop of M9 buffer for locomotion video acquisition and analysis.

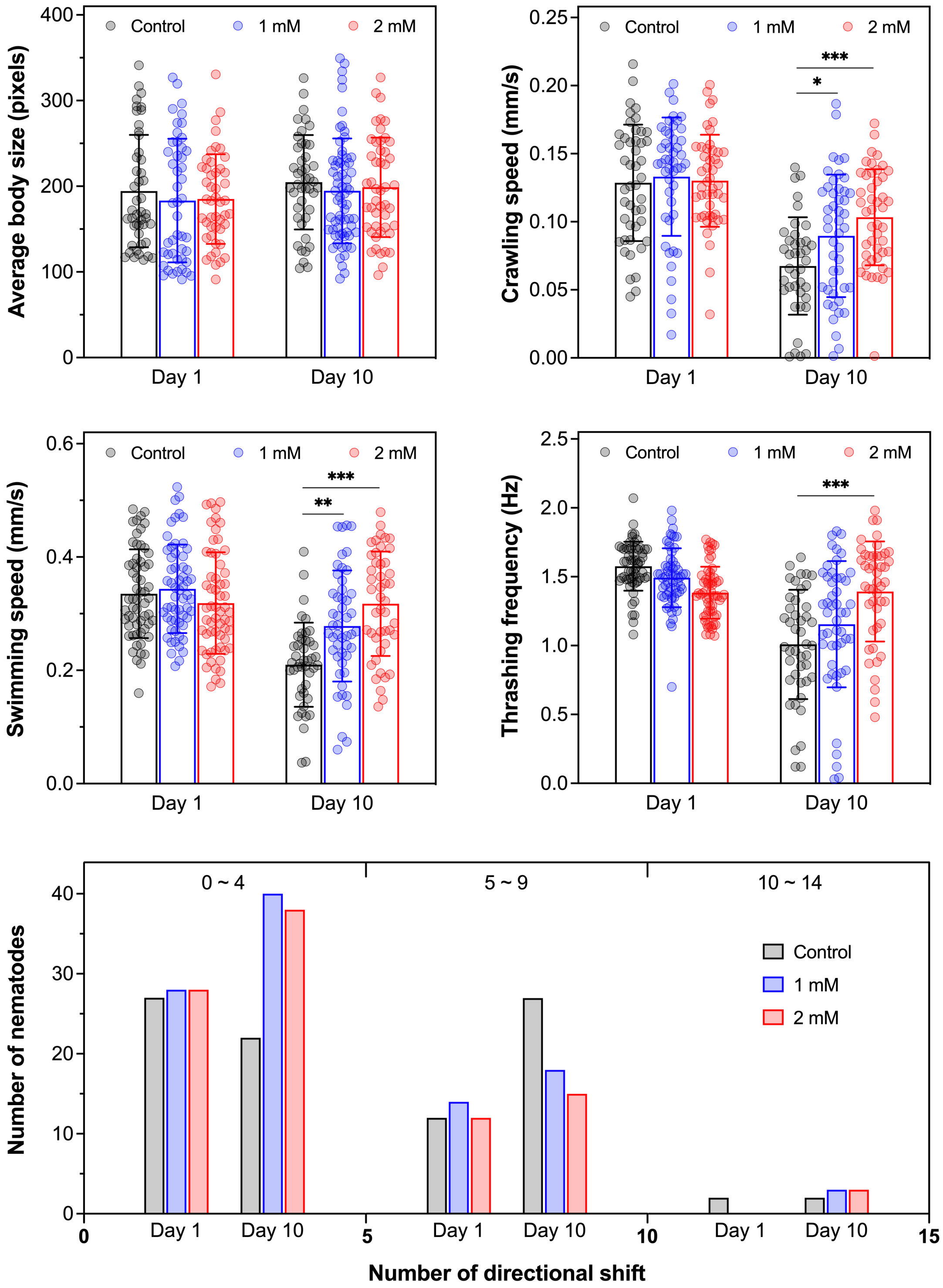

FIGURE S6 Effect of abalone peptide on fitness metrics of *C. elegans*. Wild-type nematodes were treated with AbaPep#07 from L4 until day 1 or day 10 of adulthood as indicated prior to fitness analysis. Body size and motility were analyzed as described in Figure 3. Representative results are shown as bar and scatter plots with mean ± SD from at least three independent experiments. * $p<0.05$; ** $p<0.01$; *** $p<0.001$.

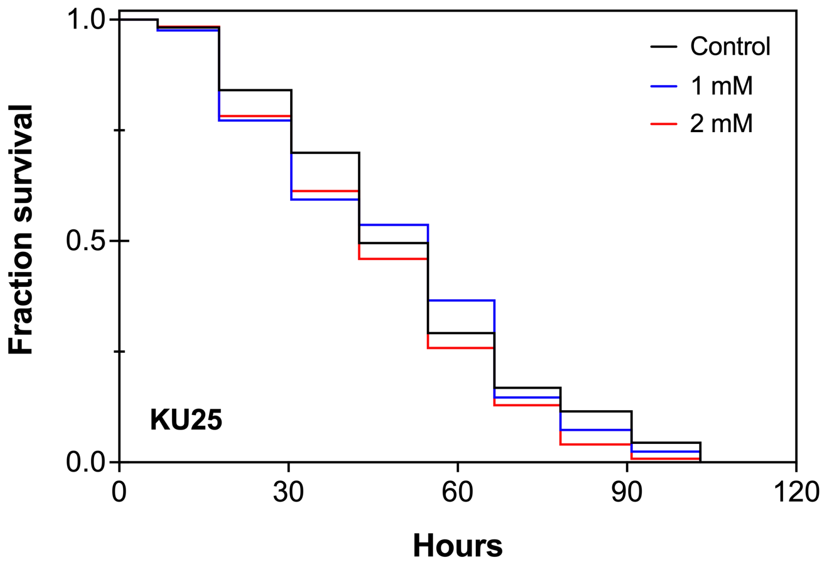

FIGURE S7 Effect of abalone peptide on survival of *pmk-1* mutant *C. elegans* against oxidative stress. The *pmk-1*(*km25*) mutant nematodes (strain KU25) were treated from L4 stage with the peptide AbaPep#07 at the indicated concentrations for 24 h and then exposed to 50 mM paraquat. Live and dead nematodes were scored every 12 h until all dead. Representative results from three independent experiments are shown as Kaplan-Meier survival curves. See Table S6 for survival statistics.

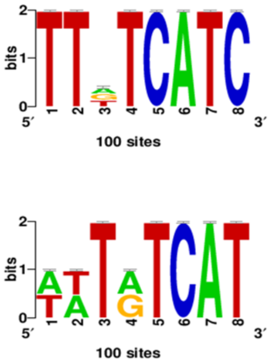

FIGURE S8 Consensus SKN-1-binding motifs. Two consensus sequences of predicted SKN-1 binding sites in *C. elegans*. RSAT and WebLogo are used to identify binding motifs and generate graphs, respectively. See also Table S8.

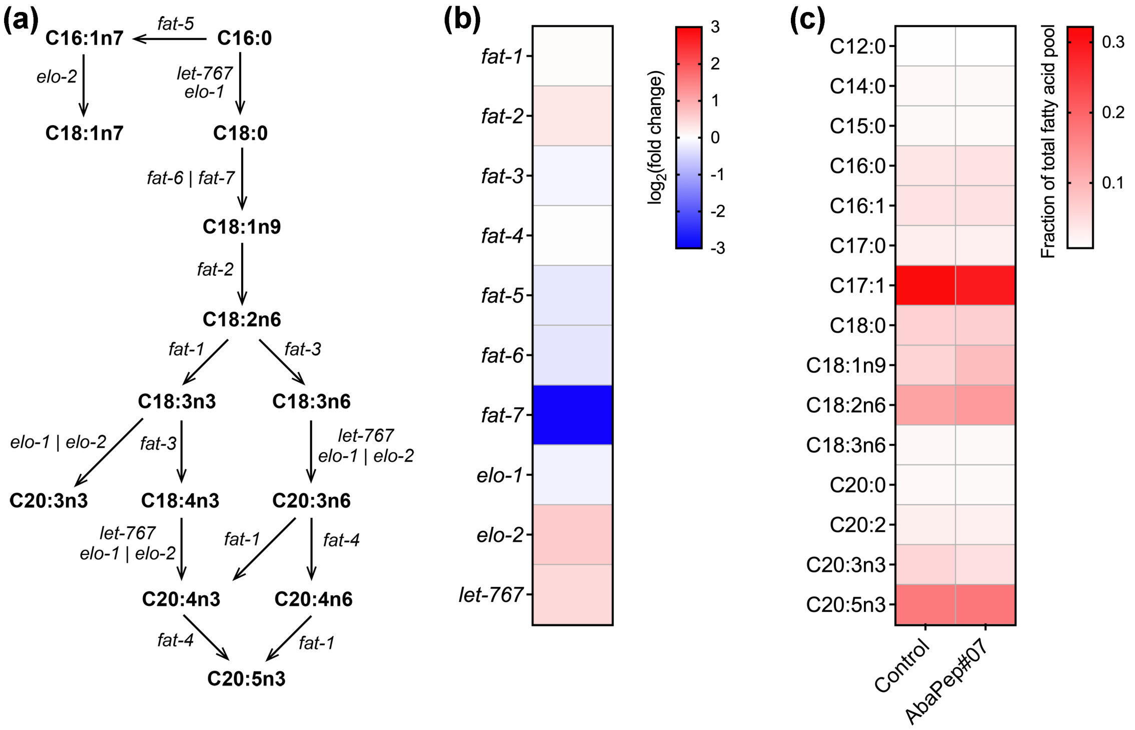

FIGURE S9 Effect of abalone peptide on expression of lipogenesis genes and composition of fatty acids in *C. elegans*. (a) Schematic of lipogenesis pathway in *C. elegans*. (b) Effect of peptide AbaPep#07 on expression of fat synthesis genes. Except *fat-7*, most lipogenesis genes were not significantly regulated by AbaPep#07. Data derived from RNA-seq are shown as a heat map. (c) Profiling of individual fatty acid fraction relative to the total fatty acid pool in *C. elegans*. Wild-type nematodes were incubated with or without AbaPep#07 from L4 to day 10 of adulthood and harvested for fatty acid analysis by GC–MS.

TABLE S1 Survival resilience of *C. elegans* to exogenous oxidative stress induced by paraquat. Stress span is expressed as the area under the curve (AUC) of lifelong survival curves of wild-type *C. elegans* populations exposed to the indicated concentration of paraquat. Life span is the AUC of lifelong survival curves of wild-type *C. elegans* populations under normal conditions in the references corresponding to the stress span. Survival resilience (S/L) is defined as the AUC ratio of stress span to life span. Related to Figure 1a.

Paraquat (mM)	Stress span (AUC)	Life span (AUC)	Survival resilience (S/L)	Reference
0.01	2400	2320	1.034	[1]
0.025	2620	2320	1.129	[1]
0.05	1582	1430	1.106	[2]
0.05	2750	2320	1.185	[1]
0.1	2575	1700	1.515	[3]
0.1	1450	1240	1.169	[4]
0.1	2660	1690	1.574	[5]
0.1	1880	1490	1.262	[6]
0.1	1806	1430	1.263	[2]
0.1	2910	2320	1.254	[1]
0.1	2450	2000	1.225	[7]
0.2	1750	1390	1.259	[8]
0.2	2890	2330	1.240	[9]
0.25	2450	1540	1.591	[10]
0.25	2410	1680	1.435	[11]
0.25	1656	1430	1.158	[2]
0.5	1310	1560	0.840	[12]
0.5	1604	1430	1.122	[2]
0.75	1444	1430	1.010	[2]
1	1390	1430	0.972	[2]
1	930	1260	0.738	[13]
2	1040	2240	0.464	[14]
2	680	1525	0.446	[15]
2	820	1700	0.482	[16]
2	637.5	2125	0.300	[17]
2	360	1520	0.237	[18]

Paraquat (mM)	Stress span (AUC)	Life span (AUC)	Survival resilience (S/L)	Reference
2	1020	1340	0.761	[19]
2	437.5	1488	0.294	[20]
2	1430	1440	0.993	[21]
2	1272	1430	0.890	[2]
2.5	1488	2213	0.672	[22]
4	490	1480	0.331	[23]
4	362.5	1688	0.215	[24]
4	1130	1730	0.653	[25]
4	412.5	1680	0.246	[26]
4	630	1680	0.375	[11]
4	570	1860	0.306	[27]
4	570	1340	0.425	[19]
4	213	1930	0.110	[28]
4	860	1560	0.551	[29]
4	775	1540	0.503	[30]
4	312	1595	0.196	[31]
5	580	1870	0.310	[32]
5	687.5	1250	0.550	[33]
5	650	1690	0.385	[34]
5	630	2330	0.270	[9]
5	600	1740	0.345	[35]
5	1170	1560	0.750	[12]
5	640	1410	0.454	[36]
5	800	1870	0.428	[37]
5	1520	1750	0.869	[38]
5	1150	1520	0.757	[39]
8	540	1690	0.320	[40]
8	886	1800	0.492	[41]
10	430	1650	0.261	[42]
10	300	1863	0.161	[43]
10	380	1700	0.224	[44]
10	535	1900	0.282	[45]
10	70.84	1288	0.055	[46]
10	912.5	2075	0.440	[47]

Paraquat (mM)	Stress span (AUC)	Life span (AUC)	Survival resilience (S/L)	Reference
10	310	1850	0.168	[48]
10	380	1730	0.220	[49]
10	376	2080	0.181	[50]
10	272	2100	0.130	[51]
10	325	1675	0.194	[52]
16	387.5	1638	0.237	[53]
20	226	1680	0.135	[54]
20	177.4	2100	0.084	[55]
20	440	2060	0.214	[56]
20	775	1638	0.473	[57]
20	260	2150	0.121	[58]
20	154.6	1250	0.124	[59]
20	330	1993	0.166	[60]
20	154.1	1930	0.080	[61]
20	358.2	2265	0.158	[62]
25	99.3	1050	0.095	[63]
30	325	1750	0.186	[64]
40	57.5	1700	0.034	[65]
40	180.6	1625	0.111	[66]
40	314	1520	0.207	[67]
40	195	1700	0.115	[68]
50	475.3	1700	0.280	[69]
50	233.8	1650	0.142	[70]
50	126.6	1763	0.072	[71]
50	475	1763	0.269	[72]
50	453.8	3150	0.144	[73]
50	477.5	1805	0.265	[74]
50	41.15	2038	0.020	[75]
50	96	1840	0.052	[76]
60	30.33	1730	0.018	[77]
60	52.7	1350	0.039	[78]
70	227.7	2100	0.108	[79]
70	231.3	2100	0.110	[80]
70	516.3	2550	0.202	[81]

Paraquat (mM)	Stress span (AUC)	Life span (AUC)	Survival resilience (S/L)	Reference
70	139.6	2070	0.067	[82]
70	178.2	2720	0.066	[83]
80	388	1430	0.271	[84]
80	270	1838	0.147	[85]
100	170	1910	0.089	[86]
100	83.33	1750	0.048	[87]
100	91.7	1460	0.063	[88]
100	100	1530	0.065	[89]
100	31.66	1360	0.023	[90]
100	171.6	1940	0.088	[86]
100	46.3	1570	0.029	[91]
100	92	1960	0.047	[92]

References for Table S1:

[1] Desjardins, D., Cacho-Valadez, B., Liu, J. L., Wang, Y., Yee, C., Bernard, K., Khaki, A., Breton, L., & Hekimi, S. (2017). Antioxidants reveal an inverted U-shaped dose-response relationship between reactive oxygen species levels and the rate of aging in *Caenorhabditis elegans*. *Aging Cell*, *16*(1), 104–112. https://doi.org/10.1111/acel.12528

[2] Meng, J., Lv, Z., Wang, Y., & Chen, C. (2022). Identification of the redox-stress signaling threshold (RST): Increased RST helps to delay aging in *C. elegans*. *Free Radical Biology & Medicine*, *178*, 54–58. https://doi.org/10.1016/j.freeradbiomed.2021.11.018

[3] Yee, C., Yang, W., & Hekimi, S. (2014). The intrinsic apoptosis pathway mediates the pro-longevity response to mitochondrial ROS in *C. elegans*. *Cell*, *157*(4), 897–909. https://doi.org/10.1016/j.cell.2014.02.055

[4] Abergel, R., Livshits, L., Shaked, M., Chatterjee, A. K., & Gross, E. (2017). Synergism between soluble guanylate cyclase signaling and neuropeptides extends lifespan in the nematode *Caenorhabditis elegans*. *Aging Cell*, *16*(2), 401–413. https://doi.org/10.1111/acel.12569

[5] Yang, W., & Hekimi, S. (2010). A mitochondrial superoxide signal triggers increased longevity in *Caenorhabditis elegans*. *PLoS Biology*, *8*(12), e1000556. https://doi.org/10.1371/journal.pbio.1000556

[6] Herholz, M., Cepeda, E., Baumann, L., Kukat, A., Hermeling, J., Maciej, S., Szczepanowska, K., Pavlenko, V., Frommolt, P., & Trifunovic, A. (2019). KLF-1 orchestrates a xenobiotic detoxification program essential for longevity of mitochondrial mutants. *Nature Communications*, *10*(1), 3323. https://doi.org/10.1038/s41467-019-11275-w

[7] Schaar, C. E., Dues, D. J., Spielbauer, K. K., Machiela, E., Cooper, J. F., Senchuk, M.,

Hekimi, S., & Van Raamsdonk, J. M. (2015). Mitochondrial and cytoplasmic ROS have opposing effects on lifespan. *PLoS Genetics*, *11*(2), e1004972. https://doi.org/10.1371/journal.pgen.1004972

[8] Gusarov, I., Pani, B., Gautier, L., Smolentseva, O., Eremina, S., Shamovsky, I., Katkova-Zhukotskaya, O., Mironov, A., & Nudler, E. (2017). Glycogen controls *Caenorhabditis elegans* lifespan and resistance to oxidative stress. *Nature Communications*, *8*, 15868. https://doi.org/10.1038/ncomms15868

[9] Xiong, L. G., Chen, Y. J., Tong, J. W., Gong, Y. S., Huang, J. A., & Liu, Z. H. (2018). Epigallocatechin-3-gallate promotes healthy lifespan through mitohormesis during early-to-mid adulthood in *Caenorhabditis elegans*. *Redox Biology*, *14*, 305–315. https://doi.org/10.1016/j.redox.2017.09.019

[10] Lee, S. J., Hwang, A. B., & Kenyon, C. (2010). Inhibition of respiration extends *C. elegans* life span via reactive oxygen species that increase HIF-1 activity. *Current Biology*, *20*(23), 2131–2136. https://doi.org/10.1016/j.cub.2010.10.057

[11] Hwang, A. B., Ryu, E. A., Artan, M., Chang, H. W., Kabir, M. H., Nam, H. J., Lee, D., Yang, J. S., Kim, S., Mair, W. B., Lee, C., Lee, S. S., & Lee, S. J. (2014). Feedback regulation via AMPK and HIF-1 mediates ROS-dependent longevity in *Caenorhabditis elegans*. *Proceedings of the National Academy of Sciences of the United States of America*, *111*(42), E4458–E4467. https://doi.org/10.1073/pnas.1411199111

[12] Zhang, M., Li, Z., Gao, D., Gong, W., Gao, Y., & Zhang, C. (2020). Hydrogen extends *Caenorhabditis elegans* longevity by reducing reactive oxygen species. *PloS One*, *15*(4), e0231972. https://doi.org/10.1371/journal.pone.0231972

[13] Yan, F., Chen, Y., Azat, R., & Zheng, X. (2017). Mulberry anthocyanin extract ameliorates oxidative damage in HepG2 cells and prolongs the lifespan of *Caenorhabditis elegans* through MAPK and Nrf2 pathways. *Oxidative Medicine and Cellular Longevity*, *2017*, 7956158. https://doi.org/10.1155/2017/7956158

[14] Keaney, M., Matthijssens, F., Sharpe, M., Vanfleteren, J., & Gems, D. (2004). Superoxide dismutase mimetics elevate superoxide dismutase activity *in vivo* but do not retard aging in the nematode *Caenorhabditis elegans*. *Free Radical Biology & Medicine*, *37*(2), 239–250. https://doi.org/10.1016/j.freeradbiomed.2004.04.005

[15] Guan, S., Li, P., Luo, J., Li, Y., Huang, L., Wang, G., Zhu, L., Fan, H., Li, W., & Wang, L. (2010). A deuterohemin peptide extends lifespan and increases stress resistance in *Caenorhabditis elegans*. *Free Radical Research*, *44*(7), 813–820. https://doi.org/10.3109/10715762.2010.485991

[16] Dues, D. J., Schaar, C. E., Johnson, B. K., Bowman, M. J., Winn, M. E., Senchuk, M. M., & Van Raamsdonk, J. M. (2017). Uncoupling of oxidative stress resistance and lifespan in long-lived *isp-1* mitochondrial mutants in *Caenorhabditis elegans*. *Free Radical Biology & Medicine*, *108*, 362–373. https://doi.org/10.1016/j.freeradbiomed.2017.04.004

[17] Artal-Sanz, M., & Tavernarakis, N. (2009). Prohibitin couples diapause signalling to

mitochondrial metabolism during ageing in *C. elegans*. *Nature*, *461*(7265), 793–797. https://doi.org/10.1038/nature08466

[18] Princz, A., Pelisch, F., & Tavernarakis, N. (2020). SUMO promotes longevity and maintains mitochondrial homeostasis during ageing in *Caenorhabditis elegans*. *Scientific Reports*, *10*(1), 15513. https://doi.org/10.1038/s41598-020-72637-9

[19] Dues, D. J., Andrews, E. K., Schaar, C. E., Bergsma, A. L., Senchuk, M. M., & Van Raamsdonk, J. M. (2016). Aging causes decreased resistance to multiple stresses and a failure to activate specific stress response pathways. *Aging*, *8*(4), 777–795. https://doi.org/10.18632/aging.100939

[20] Xu, J., Guo, Y., Sui, T., Wang, Q., Zhang, Y., Zhang, R., Wang, M., Guan, S., & Wang, L. (2017). Molecular mechanisms of anti-oxidant and anti-aging effects induced by convallatoxin in *Caenorhabditis elegans*. *Free Radical Research*, *51*(5), 529–544. https://doi.org/10.1080/10715762.2017.1331037

[21] Cooper, J. F., Machiela, E., Dues, D. J., Spielbauer, K. K., Senchuk, M. M., & Van Raamsdonk, J. M. (2017). Activation of the mitochondrial unfolded protein response promotes longevity and dopamine neuron survival in Parkinson's disease models. *Scientific Reports*, *7*(1), 16441. https://doi.org/10.1038/s41598-017-16637-2

[22] Garcia, G., Bar-Ziv, R., Averbukh, M., Dasgupta, N., Dutta, N., Zhang, H., Fan, W., Moaddeli, D., Tsui, C. K., Castro Torres, T., Alcala, A., Moehle, E. A., Hoang, S., Shalem, O., Adams, P. D., Thorwald, M. A., & Higuchi-Sanabria, R. (2023). Large-scale genetic screens identify BET-1 as a cytoskeleton regulator promoting actin function and life span. *Aging Cell*, *22*(1), e13742. https://doi.org/10.1111/acel.13742

[23] Mouchiroud, L., Houtkooper, R. H., Moullan, N., Katsyuba, E., Ryu, D., Cantó, C., Mottis, A., Jo, Y. S., Viswanathan, M., Schoonjans, K., Guarente, L., & Auwerx, J. (2013). The NAD^+/Sirtuin pathway modulates longevity through activation of mitochondrial UPR and FOXO signaling. *Cell*, *154*(2), 430–441. https://doi.org/10.1016/j.cell.2013.06.016

[24] Qian, H., Xu, X., & Niklason, L. E. (2015). PCH-2 regulates *Caenorhabditis elegans* lifespan. *Aging*, *7*(1), 1–13. https://doi.org/10.18632/aging.100713

[25] Van Raamsdonk, J. M., & Hekimi, S. (2009). Deletion of the mitochondrial superoxide dismutase *sod-2* extends lifespan in *Caenorhabditis elegans*. *PLoS Genetics*, *5*(2), e1000361. https://doi.org/10.1371/journal.pgen.1000361

[26] Ryu, D., Mouchiroud, L., Andreux, P. A., Katsyuba, E., Moullan, N., Nicolet-Dit-Félix, A. A., Williams, E. G., Jha, P., Lo Sasso, G., Huzard, D., Aebischer, P., Sandi, C., Rinsch, C., & Auwerx, J. (2016). Urolithin A induces mitophagy and prolongs lifespan in *C. elegans* and increases muscle function in rodents. *Nature Medicine*, *22*(8), 879–888. https://doi.org/10.1038/nm.4132

[27] Harris-Gauthier, N., Traa, A., AlOkda, A., Moldakozhayev, A., Anglas, U., Soo, S. K., & Van Raamsdonk, J. M. (2022). Mitochondrial thioredoxin system is required for enhanced stress resistance and extended longevity in long-lived mitochondrial mutants. *Redox*

Biology, *53*, 102335. https://doi.org/10.1016/j.redox.2022.102335

[28] Vatner, D. E., Zhang, J., Oydanich, M., Guers, J., Katsyuba, E., Yan, L., Sinclair, D., Auwerx, J., & Vatner, S. F. (2018). Enhanced longevity and metabolism by brown adipose tissue with disruption of the regulator of G protein signaling 14. *Aging Cell*, *17*(4), e12751. https://doi.org/10.1111/acel.12751

[29] Cooper, J. F., Spielbauer, K. K., Senchuk, M. M., Nadarajan, S., Colaiácovo, M. P., & Van Raamsdonk, J. M. (2018). α-Synuclein expression from a single copy transgene increases sensitivity to stress and accelerates neuronal loss in genetic models of Parkinson's disease. *Experimental Neurology*, *310*, 58–69. https://doi.org/10.1016/j.expneurol.2018.09.001

[30] Katsyuba, E., Mottis, A., Zietak, M., De Franco, F., van der Velpen, V., Gariani, K., Ryu, D., Cialabrini, L., Matilainen, O., Liscio, P., Giacchè, N., Stokar-Regenscheit, N., Legouis, D., de Seigneux, S., Ivanisevic, J., Raffaelli, N., Schoonjans, K., Pellicciari, R., & Auwerx, J. (2018). De novo NAD^+ synthesis enhances mitochondrial function and improves health. *Nature*, *563*(7731), 354–359. https://doi.org/10.1038/s41586-018-0645-6

[31] Possik, E., Jalali, Z., Nouët, Y., Yan, M., Gingras, M. C., Schmeisser, K., Panaite, L., Dupuy, F., Kharitidi, D., Chotard, L., Jones, R. G., Hall, D. H., & Pause, A. (2014). Folliculin regulates AMPK-dependent autophagy and metabolic stress survival. *PLoS Genetics*, *10*(4), e1004273. https://doi.org/10.1371/journal.pgen.1004273

[32] Shen, P., Yue, Y., Sun, Q., Kasireddy, N., Kim, K. H., & Park, Y. (2017). Piceatannol extends the lifespan of *Caenorhabditis elegans* via DAF-16. *BioFactors*, *43*(3), 379–387. https://doi.org/10.1002/biof.1346

[33] Hahm, J. H., Kim, S., & Paik, Y. K. (2009). Endogenous cGMP regulates adult longevity via the insulin signaling pathway in *Caenorhabditis elegans*. *Aging Cell*, *8*(4), 473–483. https://doi.org/10.1111/j.1474-9726.2009.00495.x

[34] Li, X., Li, J., Zhu, D., Zhang, N., Hao, X., Zhang, W., Zhang, Q., Liu, Y., Wu, X., & Tian, Y. (2022). Protein disulfide isomerase PDI-6 regulates Wnt secretion to coordinate inter-tissue UPRmt activation and lifespan extension in *C. elegans*. *Cell Reports*, *39*(10), 110931. https://doi.org/10.1016/j.celrep.2022.110931

[35] Wan, Q. L., Fu, X., Dai, W., Yang, J., Luo, Z., Meng, X., Liu, X., Zhong, R., Yang, H., & Zhou, Q. (2020). Uric acid induces stress resistance and extends the life span through activating the stress response factor DAF-16/FOXO and SKN-1/NRF2. *Aging*, *12*(3), 2840–2856. https://doi.org/10.18632/aging.102781

[36] Zhang, J., Xue, X., Qiao, Y., Li, D., Wei, Q., Zhang, F., & Qin, X. (2021). Astragaloside IV extends lifespan of *Caenorhabditis elegans* by improving age-related functional declines and triggering antioxidant responses. *Rejuvenation Research*, *24*(2), 120–130. https://doi.org/10.1089/rej.2020.2312

[37] Tawo, R., Pokrzywa, W., Kevei, É., Akyuz, M. E., Balaji, V., Adrian, S., Höhfeld, J., & Hoppe, T. (2017). The ubiquitin ligase CHIP integrates proteostasis and aging by

regulation of insulin receptor turnover. *Cell*, *169*(3), 470–482.e13. https://doi.org/10.1016/j.cell.2017.04.003

[38] Dilberger, B., Baumanns, S., Schmitt, F., Schmiedl, T., Hardt, M., Wenzel, U., & Eckert, G. P. (2019). Mitochondrial oxidative stress impairs energy metabolism and reduces stress resistance and longevity of *C. elegans*. *Oxidative Medicine and Cellular Longevity*, *2019*, 6840540. https://doi.org/10.1155/2019/6840540

[39] Kato, M., Kashem, M. A., & Cheng, C. (2016). An intestinal microRNA modulates the homeostatic adaptation to chronic oxidative stress in *C. elegans*. *Aging*, *8*(9), 1979–2005. https://doi.org/10.18632/aging.101029

[40] Zhou, B., Kreuzer, J., Kumsta, C., Wu, L., Kamer, K. J., Cedillo, L., Zhang, Y., Li, S., Kacergis, M. C., Webster, C. M., Fejes-Toth, G., Naray-Fejes-Toth, A., Das, S., Hansen, M., Haas, W., & Soukas, A. A. (2019). Mitochondrial permeability uncouples elevated autophagy and lifespan extension. *Cell*, *177*(2), 299–314.e16. https://doi.org/10.1016/j.cell.2019.02.013

[41] Yang, F., Gao, Y., Li, Z., Chen, L., Xia, Z., Xu, T., & Qin, Y. (2014). Mitochondrial EF4 links respiratory dysfunction and cytoplasmic translation in *Caenorhabditis elegans*. *Biochimica et Biophysica Acta*, *1837*(10), 1674–1683. https://doi.org/10.1016/j.bbabio.2014.05.353

[42] Deng, Y., Liu, H., Huang, Q., Tu, L., Hu, L., Zheng, B., Sun, H., Lu, D., Guo, C., & Zhou, L. (2022). Mechanism of longevity extension of *Caenorhabditis elegans* induced by *Schizophyllum commune* fermented supernatant with added Radix Puerariae. *Frontiers in Nutrition*, *9*, 847064. https://doi.org/10.3389/fnut.2022.847064

[43] Zarse, K., Schmeisser, S., Groth, M., Priebe, S., Beuster, G., Kuhlow, D., Guthke, R., Platzer, M., Kahn, C. R., & Ristow, M. (2012). Impaired insulin/IGF1 signaling extends life span by promoting mitochondrial L-proline catabolism to induce a transient ROS signal. *Cell Metabolism*, *15*(4), 451–465. https://doi.org/10.1016/j.cmet.2012.02.013

[44] Urban, N., Tsitsipatis, D., Hausig, F., Kreuzer, K., Erler, K., Stein, V., Ristow, M., Steinbrenner, H., & Klotz, L. O. (2017). Non-linear impact of glutathione depletion on *C. elegans* life span and stress resistance. *Redox Biology*, *11*, 502–515. https://doi.org/10.1016/j.redox.2016.12.003

[45] Lin, C., Zhang, X., Su, Z., Xiao, J., Lv, M., Cao, Y., & Chen, Y. (2019). Carnosol improved lifespan and healthspan by promoting antioxidant capacity in *Caenorhabditis elegans*. *Oxidative Medicine and Cellular Longevity*, *2019*, 5958043. https://doi.org/10.1155/2019/5958043

[46] Bao, K., Liu, W., Song, Z., Feng, J., Mao, Z., Bao, L., Sun, T., Hu, Z., & Li, J. (2022). Crotamiton derivative JM03 extends lifespan and improves oxidative and hypertonic stress resistance in *Caenorhabditis elegans* via inhibiting OSM-9. *eLife*, 11, e72410. https://doi.org/10.7554/eLife.72410

[47] Li, N., Li, X., Shi, Y. L., Gao, J. M., He, Y. Q., Li, F., Shi, J. S., & Gong, Q. H. (2021).

Trilobatin, a component from *Lithocarpus polystachyrus* Rehd., increases longevity in *C. elegans* through activating SKN1/SIRT3/DAF16 signaling pathway. *Frontiers in Pharmacology*, *12*, 655045. https://doi.org/10.3389/fphar.2021.655045

[48] Lin, C., Chen, Y., Lin, Y., Wang, X., Hu, L., Cao, Y., & Chen, Y. (2021). Antistress and anti-aging activities of *Caenorhabditis elegans* were enhanced by *Momordica saponin* extract. *European Journal of Nutrition*, *60*(4), 1819–1832. https://doi.org/10.1007/s00394-020-02338-6

[49] Zimmerman, S. M., & Kim, S. K. (2014). The GATA transcription factor/MTA-1 homolog *egr-1* promotes longevity and stress resistance in *Caenorhabditis elegans*. *Aging Cell*, *13*(2), 329–339. https://doi.org/10.1111/acel.12179

[50] Philipp, T. M., Gong, W., Köhnlein, K., Ohse, V. A., Müller, F. I., Priebs, J., Steinbrenner, H., & Klotz, L. O. (2022). SEMO-1, a novel methanethiol oxidase in *Caenorhabditis elegans*, is a pro-aging factor conferring selective stress resistance. *BioFactors*, *48*(3), 699–706. https://doi.org/10.1002/biof.1836

[51] Zarse, K., Jabin, S., & Ristow, M. (2012). L-Theanine extends lifespan of adult *Caenorhabditis elegans*. *European Journal of Nutrition*, *51*(6), 765–768. https://doi.org/10.1007/s00394-012-0341-5

[52] Aman, Y., Erinjeri, A. P., Tataridas-Pallas, N., Williams, R., Wellman, R., Chapman, H., & Labbadia, J. (2022). Loss of MTCH-1 suppresses age-related proteostasis collapse through the inhibition of programmed cell death factors. *Cell Reports*, *41*(8), 111690. https://doi.org/10.1016/j.celrep.2022.111690

[53] Walter, L., Baruah, A., Chang, H. W., Pace, H. M., & Lee, S. S. (2011). The homeobox protein CEH-23 mediates prolonged longevity in response to impaired mitochondrial electron transport chain in *C. elegans*. *PLoS Biology*, *9*(6), e1001084. https://doi.org/10.1371/journal.pbio.1001084

[54] Seo, K., Choi, E., Lee, D., Jeong, D. E., Jang, S. K., & Lee, S. J. (2013). Heat shock factor 1 mediates the longevity conferred by inhibition of TOR and insulin/IGF-1 signaling pathways in *C. elegans*. *Aging Cell*, *12*(6), 1073–1081. https://doi.org/10.1111/acel.12140

[55] Wei, Y., & Kenyon, C. (2016). Roles for ROS and hydrogen sulfide in the longevity response to germline loss in *Caenorhabditis elegans*. *Proceedings of the National Academy of Sciences of the United States of America*, *113*(20), E2832–E2841. https://doi.org/10.1073/pnas.1524727113

[56] Lu, M., Tan, L., Zhou, X. G., Yang, Z. L., Zhu, Q., Chen, J. N., Luo, H. R., & Wu, G. S. (2020). Secoisolariciresinol diglucoside delays the progression of aging-related diseases and extends the lifespan of *Caenorhabditis elegans* via DAF-16 and HSF-1. *Oxidative Medicine and Cellular Longevity*, *2020*, 1293935. https://doi.org/10.1155/2020/1293935

[57] Liu, Y., Lu, Y. Y., Huang, L., Shi, L., Zheng, Z. Y., Chen, J. N., Qu, Y., Xiao, H. T., Luo, H. R., & Wu, G. S. (2022). *Para*-hydroxybenzyl alcohol delays the progression of

neurodegenerative diseases in models of *Caenorhabditis elegans* through activating multiple cellular protective pathways. *Oxidative Medicine and Cellular Longevity*, *2022*, 8986287. https://doi.org/10.1155/2022/8986287

[58] Roy, C., Molin, L., Alcolei, A., Solyga, M., Bonneau, B., Vachon, C., Bessereau, J. L., & Solari, F. (2022). DAF-2/insulin IGF-1 receptor regulates motility during aging by integrating opposite signaling from muscle and neuronal tissues. *Aging Cell*, *21*(8), e13660. https://doi.org/10.1111/acel.13660

[59] Kew, C., Huang, W., Fischer, J., Ganesan, R., Robinson, N., & Antebi, A. (2020). Evolutionarily conserved regulation of immunity by the splicing factor RNP-6/PUF60. *eLife*, *9*, e57591. https://doi.org/10.7554/eLife.57591

[60] Ranjan, M., Gruber, J., Ng, L. F., & Halliwell, B. (2013). Repression of the mitochondrial peroxiredoxin antioxidant system does not shorten life span but causes reduced fitness in *Caenorhabditis elegans*. *Free Radical Biology & Medicine*, *63*, 381–389. https://doi.org/10.1016/j.freeradbiomed.2013.05.025

[61] Admasu, T. D., Barardo, D., Ng, L. F., Batchu, K. C., Cazenave-Gassiot, A., Wenk, M. R., & Gruber, J. (2022). A small-molecule Psora-4 acts as a caloric restriction mimetic to promote longevity in *C. elegans*. *GeroScience*, *44*(2), 1029–1046. https://doi.org/10.1007/s11357-021-00374-6

[62] Park, S. K., Link, C. D., & Johnson, T. E. (2010). Life-span extension by dietary restriction is mediated by NLP-7 signaling and coelomocyte endocytosis in *C. elegans*. *FASEB Journal*, *24*(2), 383–392. https://doi.org/10.1096/fj.09-142984

[63] Wu, M., Kang, X., Wang, Q., Zhou, C., Mohan, C., & Peng, A. (2017). Regulator of G protein signaling-1 modulates paraquat-induced oxidative stress and longevity via the insulin like signaling pathway in *Caenorhabditis elegans*. *Toxicology Letters*, *273*, 97–105. https://doi.org/10.1016/j.toxlet.2017.03.027

[64] Huang, Q., Li, R., Yi, T., Cong, F., Wang, D., Deng, Z., & Zhao, Y. L. (2021). Phosphorothioate-DNA bacterial diet reduces the ROS levels in *C. elegans* while improving locomotion and longevity. *Communications Biology*, *4*(1), 1335. https://doi.org/10.1038/s42003-021-02863-y

[65] Doonan, R., McElwee, J. J., Matthijssens, F., Walker, G. A., Houthoofd, K., Back, P., Matscheski, A., Vanfleteren, J. R., & Gems, D. (2008). Against the oxidative damage theory of aging: superoxide dismutases protect against oxidative stress but have little or no effect on life span in *Caenorhabditis elegans*. *Genes & Development*, *22*(23), 3236–3241. https://doi.org/10.1101/gad.504808

[66] Kumar, S., Dietrich, N., & Kornfeld, K. (2016). Angiotensin converting enzyme (ACE) inhibitor extends *Caenorhabditis elegans* life span. *PLoS Genetics*, *12*(2), e1005866. https://doi.org/10.1371/journal.pgen.1005866

[67] Taylor, R. C., & Dillin, A. (2013). XBP-1 is a cell-nonautonomous regulator of stress resistance and longevity. *Cell*, *153*(7), 1435–1447. https://doi.org/10.1016/j.cell.

2013.05.042

[68] Warnhoff, K., Murphy, J. T., Kumar, S., Schneider, D. L., Peterson, M., Hsu, S., Guthrie, J., Robertson, J. D., & Kornfeld, K. (2014). The DAF-16 FOXO transcription factor regulates *natc-1* to modulate stress resistance in *Caenorhabditis elegans*, linking insulin/IGF-1 signaling to protein N-terminal acetylation. *PLoS Genetics*, *10*(10), e1004703. https://doi.org/10.1371/journal.pgen.1004703

[69] Sun, M. L., Chen, X. Y., Cao, J. J., Cui, X. H., & Wang, H. B. (2021). *Polygonum multiflorum* Thunb extract extended the lifespan and healthspan of *Caenorhabditis elegans* via DAF-16/SIR-2.1/SKN-1. *Food & Function*, *12*(18), 8774–8786. https://doi.org/10.1039/d1fo01908b

[70] Schulz, T. J., Zarse, K., Voigt, A., Urban, N., Birringer, M., & Ristow, M. (2007). Glucose restriction extends *Caenorhabditis elegans* life span by inducing mitochondrial respiration and increasing oxidative stress. *Cell Metabolism*, *6*(4), 280–293. https://doi.org/10.1016/j.cmet.2007.08.011

[71] Mori, M. A., Raghavan, P., Thomou, T., Boucher, J., Robida-Stubbs, S., Macotela, Y., Russell, S. J., Kirkland, J. L., Blackwell, T. K., & Kahn, C. R. (2012). Role of microRNA processing in adipose tissue in stress defense and longevity. *Cell Metabolism*, *16*(3), 336–347. https://doi.org/10.1016/j.cmet.2012.07.017

[72] Li, H., Yu, X., Li, C., Ma, L., Zhao, Z., Guan, S., & Wang, L. (2021). Caffeic acid protects against Aβ toxicity and prolongs lifespan in *Caenorhabditis elegans* models. *Food & Function*, *12*(3), 1219–1231. https://doi.org/10.1039/d0fo02784g

[73] Guo, K., Su, L., Wang, Y., Liu, H., Lin, J., Cheng, P., Yin, X., Liang, M., Wang, Q., & Huang, Z. (2020). Antioxidant and anti-aging effects of a sea cucumber protein hydrolyzate and bioinformatic characterization of its composing peptides. *Food & Function*, *11*(6), 5004–5016. https://doi.org/10.1039/d0fo00560f

[74] Ke, J. P., Yu, J. Y., Gao, B., Hu, F. L., Xu, F. Q., Yao, G., & Bao, G. H. (2022). Two new catechins from Zijuan green tea enhance the fitness and lifespan of *Caenorhabditis elegans* via insulin-like signaling pathways. *Food & Function*, *13*(18), 9299–9310. https://doi.org/10.1039/d2fo01795d

[75] Tharp, K. M., Higuchi-Sanabria, R., Timblin, G. A., Ford, B., Garzon-Coral, C., Schneider, C., Muncie, J. M., Stashko, C., Daniele, J. R., Moore, A. S., Frankino, P. A., Homentcovschi, S., Manoli, S. S., Shao, H., Richards, A. L., Chen, K. H., Hoeve, J. T., Ku, G. M., Hellerstein, M., Nomura, D. K., Saijo, K., Gestwicki, J., Dunn, A. R., Krogan, N. J., Swaney, D. L., Dillin, A., & Weaver, V. M. (2021). Adhesion-mediated mechanosignaling forces mitohormesis. *Cell Metabolism*, *33*(7), 1322–1341.e13. https://doi.org/10.1016/j.cmet.2021.04.017

[76] Honda, Y., & Honda, S. (2002). Life span extensions associated with upregulation of gene expression of antioxidant enzymes in *Caenorhabditis elegans*; studies of mutation in the *age-1*, PI3 kinase homologue and short-term exposure to hyperoxia. *Journal of the*

American Aging Association, *25*(1), 21–28. https://doi.org/10.1007/s11357-002-0003-2
[77] Lim, H. J., Han, Y. T., Ahn, J. H., Jeon, Y. D., Jeon, H., & Cha, D. S. (2020). Longevity effects of hispidol in *Caenorhabditis elegans*. *BioFactors*, *46*(6), 1041–1048. https://doi.org/10.1002/biof.1695
[78] Kim, Y. S., Seo, H. W., Lee, M. H., Kim, D. K., Jeon, H., & Cha, D. S. (2014). Protocatechuic acid extends lifespan and increases stress resistance in *Caenorhabditis elegans*. *Archives of Pharmacal Research*, *37*(2), 245–252. https://doi.org/10.1007/s12272-013-0183-6
[79] Wang, Q., Huang, Y., Qin, C., Liang, M., Mao, X., Li, S., Zou, Y., Jia, W., Li, H., Ma, C. W., & Huang, Z. (2016). Bioactive peptides from *Angelica sinensis* protein hydrolyzate delay senescence in *Caenorhabditis elegans* through antioxidant activities. *Oxidative Medicine and Cellular Longevity*, 2016, 8956981. https://doi.org/10.1155/2016/8956981
[80] Xiang, Y., Zhang, J., Li, H., Wang, Q., Xiao, L., Weng, H., Zhou, X., Ma, C. W., Ma, F., Hu, M., & Huang, Z. (2017). Epimedium polysaccharide alleviates polyglutamine-induced neurotoxicity in *Caenorhabditis elegans* by reducing oxidative stress. *Rejuvenation Research*, 20(1), 32–41. https://doi.org/10.1089/rej.2016.1830
[81] Ruangchuay, S., Wang, Q. Q., Wang, L. Y., Lin, J., Wang, Y. C., Zhong, G. H., Maneenoon, K., Huang, Z. B., & Chusri, S. (2021). Antioxidant and antiaging effect of traditional Thai rejuvenation medicines in *Caenorhabditis elegans*. *Journal of Integrative Medicine*, *19*(4), 362–373. https://doi.org/10.1016/j.joim.2021.03.004
[82] Ghosh, B., Guidry, H. J., Johnston, M., & Bohnert, K. A. (2022). A fat-promoting botanical extract from *Artemisia scoparia* exerts geroprotective effects on *Caenorhabditis elegans* life span and stress resistance. *The Journals of Gerontology. Series A, Biological Sciences and Medical Sciences*, *77*(6), 1112–1120. https://doi.org/10.1093/gerona/glac040
[83] Zhong, G., Pan, W., Huang, Z., Guo, K., Hu, J., Liu, P., Chen, S., Wang, Y., Ai, L., & Huang, Z. (2021). Physicochemical and geroprotective comparison of *Nostoc sphaeroides* polysaccharides across colony growth stages and with derived oligosaccharides. *Journal of Applied Phycology*, *33*(2), 939–952. https://doi.org/10.1007/s10811-021-02383-6
[84] Kim, Y., & Sun, H. (2012). ASM-3 acid sphingomyelinase functions as a positive regulator of the DAF-2/AGE-1 signaling pathway and serves as a novel anti-aging target. *PloS One*, *7*(9), e45890. https://doi.org/10.1371/journal.pone.0045890
[85] Saier, C., Storbeck, S., Baier, S., Dietz, H. & Wa ̈tjen, W. (2020). Rosemary extract modulates stress resistance and accumulation of reactive oxygen species in the model organism *Caenorhabditis elegans*. *PharmaNutrition*, *14*(2020): 100233. https://doi.org/10.1016/j.phanu.2020.100233
[86] Cuong, V. T., Chen, W., Shi, J., Zhang, M., Yang, H., Wang, N., Yang, S., Li, J., Yang, P., & Fei, J. (2019). The anti-oxidation and anti-aging effects of *Ganoderma lucidum* in

Caenorhabditis elegans. *Experimental Gerontology*, *117*, 99–105. https://doi.org/10.1016/j.exger.2018.11.016

[87] Anderson, R. T., Bradley, T. A., & Smith, D. M. (2022). Hyperactivation of the proteasome in *Caenorhabditis elegans* protects against proteotoxic stress and extends lifespan. *The Journal of Biological Chemistry*, 102415. https://doi.org/10.1016/j.jbc.2022.102415

[88] Chamoli, M., Singh, A., Malik, Y., & Mukhopadhyay, A. (2014). A novel kinase regulates dietary restriction-mediated longevity in *Caenorhabditis elegans*. *Aging Cell*, *13*(4), 641–655. https://doi.org/10.1111/acel.12218

[89] An, J. H., & Blackwell, T. K. (2003). SKN-1 links *C. elegans* mesendodermal specification to a conserved oxidative stress response. *Genes & Development*, *17*(15), 1882–1893. https://doi.org/10.1101/gad.1107803

[90] Jin, C., Li, J., Green, C. D., Yu, X., Tang, X., Han, D., Xian, B., Wang, D., Huang, X., Cao, X., Yan, Z., Hou, L., Liu, J., Shukeir, N., Khaitovich, P., Chen, C. D., Zhang, H., Jenuwein, T., & Han, J. D. (2011). Histone demethylase UTX-1 regulates *C. elegans* life span by targeting the insulin/IGF-1 signaling pathway. *Cell Metabolism*, *14*(2), 161–172. https://doi.org/10.1016/j.cmet.2011.07.001

[91] Budovskaya, Y. V., Wu, K., Southworth, L. K., Jiang, M., Tedesco, P., Johnson, T. E., & Kim, S. K. (2008). An *elt-3*/*elt-5*/*elt-6* GATA transcription circuit guides aging in *C. elegans*. *Cell*, *134*(2), 291–303. https://doi.org/10.1016/j.cell.2008.05.044

[92] Tullet, J. M. A., Green, J. W., Au, C., Benedetto, A., Thompson, M. A., Clark, E., Gilliat, A. F., Young, A., Schmeisser, K., & Gems, D. (2017). The SKN-1/Nrf2 transcription factor can protect against oxidative stress and increase lifespan in *C. elegans* by distinct mechanisms. *Aging Cell*, *16*(5), 1191–1194. https://doi.org/10.1111/acel.12627

TABLE S2 Statistics for *C. elegans* survival assays under paraquat-induced oxidative stress. The survival time is the period that the wild-type nematodes pretreated with or without protein hydrolysate/AbaPep#07 were still alive after exposure to 50 mM paraquat. Related to Figure 1b and 1c.

Repeat	Group	Survival time (h)			*p* value	Number of
		Mean ± SD	Median ± SD	Maximum	(log-rank test)	nematodes
Abalone protein hydrolysate						
#1	Control	103.8 ± 2.0	107.3 ± 1.4	202.3		142
	1 mg/mL	115.2 ± 2.3	107.3 ± 2.1	215.0	<0.001	149
	2 mg/mL	126.6 ± 2.6	120.5 ± 2.7	215.0	<0.001	143
	4 mg/mL	135.5 ± 2.9	132.0 ± 2.9	228.0	<0.001	144
#2	Control	116.3 ± 2.7	116.7 ± 3.7	200.5		124
	1 mg/mL	138.2 ± 3.2	138.8 ± 4.2	213.0	<0.001	114
	2 mg/mL	159.8 ± 4.0	165.0 ± 5.5	248.8	<0.001	115
	4 mg/mL	200.8 ± 4.7	200.5 ± 5.7	284.8	<0.001	128
#3	Control	122.2 ± 1.9	126.2 ± 1.8	163.5		130
	1 mg/mL	137.7 ± 2.0	141.0 ± 2.9	190.0	<0.001	136
	2 mg/mL	141.7 ± 2.1	151.7 ± 3.0	200.2	<0.001	129
	4 mg/mL	160.6 ± 2.8	163.5 ± 2.8	239.0	<0.001	136
Abalone peptide AbaPep#07						
#1	Control	147.7 ± 1.7	151.0 ± 3.4	223.5		279
	1 mM	198.6 ± 3.0	199.7 ± 1.7	320.6	<0.001	232
	2 mM	196.6 ± 2.6	188.7 ± 3.5	308.3	<0.001	289
#2	Control	135.8 ± 3.4	148.2 ± 6.7	194.8		138
	1 mM	192.2 ± 3.0	194.8 ± 1.1	256.4	<0.001	151
	2 mM	206.9 ± 3.0	208.7 ± 2.6	266.5	<0.001	166
#3	Control	133.4 ± 2.0	136.0 ± 1.8	194.3		283
	1 mM	168.5 ± 2.6	170.0 ± 2.1	303.3	<0.001	274
	2 mM	184.5 ± 3.8	183.8 ± 3.3	327.2	<0.001	203

TABLE S3 Peptides identified in abalone protein hydrolysate and tested for antioxidant capacity. The hydrolysate was subjected to activity-guided fractionation by ultrafiltration and gel filtration, and the peptide sequences in antioxidant fractions were identified by RP-nano-LC-MS/MS. After *in silico* analysis of the sequences for antioxidant potential using BIOPEP-UWM database, the peptides of interest were synthesized and tested for antioxidant activity using paraquat survival assay in *C. elegans*.

ID	Sequence	MW (Da)	Antioxidant indicator*	ΔAUC%#
AbaPep#01	NLWQVAAS	888.0	LWQ; LW	13.8%
AbaPep#02	TNLWQVAA	902.0	LWQ; LW	25.7%
AbaPep#03	TGTYADFM	905.0	ADF; TY	29.0%
AbaPep#04	IGVNIPWH	935.1	PWH; PW	15.8%
AbaPep#05	TNLWQVAAS	989.1	LWQ; LW	6.1%
AbaPep#06	AIGVNIPWH	1006.2	PWH; PW	30.0%
AbaPep#07	SETYELRK	1025.1	EL; TY	47.6%
AbaPep#08	TYADFMEAF	1094.2	ADF; TY	37.8%
AbaPep#09	KDLELAVISH	1124.3	KD; EL	8.7%
AbaPep#10	AIGVNIPWHK	1134.4	PWH; PW	33.3%
AbaPep#11	GTYADFMEAF	1151.2	ADF; TY	33.5%
AbaPep#12	TGTYADFMEAF	1252.3	ADF; TY	45.8%
AbaPep#13	TNLWQVAASIR	1258.4	LWQ; IR; LW	28.9%
AbaPep#14	ATNPLQSNPGTIR	1368.5	IR; PLQ	25.3%
AbaPep#15	DTGTYADFMEAFK	1495.6	ADF; TY	20.1%
AbaPep#16	VLAYEPVWAIGTGK	1503.8	AY; VW	5.0%
AbaPep#17	DVLPDYFEPWNR	1550.7	PWN; PW	21.0%
AbaPep#18	VVLAYEPVWAIGTGK	1602.9	AY; VW	15.2%
AbaPep#19	LADELKQEQENYK	1607.7	EL; LK	24.5%
AbaPep#20	NFLVWVNEEDHLR	1670.8	HL; VW	10.9%
AbaPep#21	LDSTNANCHEAHLYC	1690.8	HL; LY; AH	3.7%
AbaPep#22	ALDSTNANCHEAHLYC	1761.9	HL; LY; AH	6.5%
AbaPep#23	ALDSTNANCHEAHLYCR	1918.1	HL; LY; AH	3.2%

* Identified against a set of dipeptide and tripeptide antioxidant indicators in the BIOPEP-UWM database, which was used here for *in silico* analysis of peptide sequences.

# The relative change in area under the survival curve of *C. elegans* with *versus* without peptide treatment in paraquat survival assay, indicating a relative total survival gain or loss.

TABLE S4 Survival resilience of *C. elegans* to endogenous proteotoxic stress. Stress span is expressed as the area under the curve (AUC) of population survival curves of transgenic *C. elegans* expressing polyQ or Aβ in body wall muscle cells. Life span is the AUC of population survival curves of wild-type or transgenic control *C. elegans* (without polyQ/Aβ expression) in the references corresponding to the stress span. Survival resilience (S/L) is defined as the AUC ratio of stress span to life span.

Strain	Stress span (AUC)	Life span (AUC)	Survival resilience (S/L)	Reference
PolyQ35 expressing strains				
AM140	1225	1638	0.748	[1]
	1354	1702	0.796	[2]
	1210	2150	0.563	[3]
	1500	1780	0.843	[4]
Aβ1-42 expressing strains				
GMC101	890	1060	0.840	[5]
	1280	1810	0.707	[6]
	619	1465	0.423	[7]
CL2006	1175	2200	0.534	[8]
	1563	2613	0.598	[9]
	1175	1550	0.758	[10]
	1690	2090	0.809	[11]
	1330	2280	0.583	[12]
	1240	1610	0.770	[13]
CL2120	1100	1540	0.714	[13]

References for Table S4:

[1] Gatrell, L., Wilkins, W., Rana, P., & Farris, M. (2020). Glucose effects on polyglutamine-induced proteotoxic stress in *Caenorhabditis elegans*. *Biochemical and Biophysical Research Communications*, *522*(3), 709–715. https://doi.org/10.1016/j.bbrc.2019.11.159

[2] Haldimann, P., Muriset, M., Vígh, L., & Goloubinoff, P. (2011). The novel hydroxylamine derivative NG-094 suppresses polyglutamine protein toxicity in *Caenorhabditis elegans*. *Journal of Biological Chemistry*, *286*(21), 18784–18794. https://doi.org/10.1074/jbc.M111.234773

[3] Lin, C., Zhang, X., Zhuang, C., Lin, Y., Cao, Y., & Chen, Y. (2020). Healthspan improvements in *Caenorhabditis elegans* with traditional Chinese herbal tea. *Oxidative Medicine and Cellular Longevity*, *2020*, 4057841. https://doi.org/10.1155/2020/4057841

[4] Ma, X., Li, J., Cui, X., Li, F., & Wang, Z. (2019). Dietary supplementation with peptides

from sesame cake protect *Caenorhabditis elegans* from polyglutamine-induced toxicity. *Journal of Functional Foods*, *54*, 199–210. https://doi.org/10.1016/j.jff.2019.01.002

[5] Huang, L., Luo, Y., Pu, Z., Kong, X., Fu, X., Xing, H., Wei, S., Chen, W., & Tang, H. (2017). Oxoisoaporphine alkaloid derivative 8-1 reduces Aβ1-42 secretion and toxicity in human cell and *Caenorhabditis elegans* models of Alzheimer's disease. *Neurochemistry International*, *108*, 157–168. https://doi.org/10.1016/j.neuint.2017.03.007

[6] Huang, J., Chen, S., Hu, L., Niu, H., Sun, Q., Li, W., Tan, G., Li, J., Jin, L., Lyu, J., & Zhou, H. (2018). Mitoferrin-1 is involved in the progression of Alzheimer's disease through targeting mitochondrial iron metabolism in a *Caenorhabditis elegans* model of Alzheimer's disease. *Neuroscience*, *385*, 90–101. https://doi.org/10.1016/j.neuroscience.2018.06.011

[7] Lima, T. I., Laurila, P. P., Wohlwend, M., Morel, J. D., Goeminne, L. J. E., Li, H., Romani, M., Li, X., Oh, C. M., Park, D., Rodríguez-López, S., Ivanisevic, J., Gallart-Ayala, H., Crisol, B., Delort, F., Batonnet-Pichon, S., Silveira, L. R., Sankabattula Pavani Veera Venkata, L., Padala, A. K., Jain, S., … Auwerx, J. (2023). Inhibiting de novo ceramide synthesis restores mitochondrial and protein homeostasis in muscle aging. *Science Translational Medicine*, *15*(696), eade6509. https://doi.org/10.1126/scitranslmed.ade6509

[8] Ai, L., Yang, F., Song, J., Chen, Y., Xiao, L., Wang, Q., Wang, L., Li, H., Lei, T., & Huang, Z. (2018). Inhibition of Abeta proteotoxicity by paeoniflorin in *Caenorhabditis elegans* through regulation of oxidative and heat shock stress responses. *Rejuvenation Research*, 21(4), 304–312. https://doi.org/10.1089/rej.2017.1966

[9] Lin, S., Wei, L., Ping, Y., Xia, L., & Xiao, S. (2018). Upregulated BMP6 pathway involved in the pathogenesis of Aβ toxicity *in vivo*. *Neuroscience Letters*, *664*, 152–159. https://doi.org/10.1016/j.neulet.2017.11.022

[10] Xiao, Y., Zhang, H., Sheng, Y., Liu, F., Gao, J., Liu, G., Li, S., Jiang, N., Yu, C. & Liu, Y. (2022). Usnic acid extends healthspan and improves the neurodegeneration diseases via mTOR/PHA-4 signaling pathway in *Caenorhabditis elegans*. *iScience*, *25*(12), 105539. https://doi.org/10.1016/j.isci.2022.105539

[11] Tan, L., Zheng, Z. Y., Huang, L., Jin, Z., Li, S. L., Wu, G. S., & Luo, H. R. (2022). Flavonol glycoside complanatoside A requires FOXO/DAF-16, NRF2/SKN-1, and HSF-1 to improve stress resistances and extend the life span of *Caenorhabditis elegans*. *Frontiers in Pharmacology*, *13*, 931886. https://doi.org/10.3389/fphar.2022.931886

[12] Frandsen, J., Choi, S. R., & Narayanasamy, P. (2020). Neural glyoxalase pathway enhancement by morin derivatives in an Alzheimer's disease model. *ACS Chemical Neuroscience*, *11*(3), 356–366. https://doi.org/10.1021/acschemneuro.9b00566

[13] Cogliati, S., Clementi, V., Francisco, M., Crespo, C., Argañaraz, F., & Grau, R. (2020). *Bacillus subtilis* delays neurodegeneration and behavioral impairment in the Alzheimer's disease model *Caenorhabditis elegans*. *Journal of Alzheimer's Disease*, *73*(3), 1035–1052. https://doi.org/10.3233/JAD-190837

TABLE S5 Statistics for *C. elegans* life span assays. Life span is the days of adulthood of wild-type nematodes treated with the indicated concentrations of AbaPep#07 from L4 stage. Related to Figure 3a.

Repeat	Group	Life span (days of adulthood)			*p* value (log-rank test)	Number of nematodes
		Mean ± SD	Median ± SD	Maximum		
#1	Control	19.4 ± 0.6	20.0 ± 0.4	38.0		106
	1 mM	21.8 ± 0.7	22.0 ± 0.8	40.0	0.004	111
	2 mM	23.9 ± 0.7	22.0 ± 0.7	40.0	<0.001	120
#2	Control	18.8 ± 0.6	18.0 ± 0.7	36.0		133
	1 mM	20.0 ± 0.7	18.0 ± 0.7	42.0	0.175	116
	2 mM	22.0 ± 0.7	20.0 ± 0.6	44.0	0.001	131
#3	Control	17.1 ± 0.6	16.0 ± 0.8	34.0		120
	1 mM	20.4 ± 0.6	20.0 ± 0.7	40.0	<0.001	128
	2 mM	23.8 ± 0.9	24.0 ± 1.6	42.0	<0.001	113

TABLE S6 Statistics for survival assays of mutant *C. elegans* under paraquat-induced oxidative stress. The survival time is the period that the nematodes pretreated with or without AbaPep#07 were still alive after exposure to 50 mM paraquat. The data for *skn-1(zu67)* (strain EU1) and *pmk-1(km25)* (strain KU25) mutants are related to Figure 4c (top panel) and Figure S7, respectively.

Repeat	Group	Survival time (h)			*p* value (log-rank test)	Number of nematodes
		Mean ± SD	Median ± SD	Maximum		
Strain EU1 (*skn-1* mutant)						
#1	Control	70.0 ± 1.1	65.0 ± 3.7	119.0		137
	1 mM	68.9 ± 1.8	65.0 ± 2.5	119.0	0.505	155
	2 mM	71.2 ± 2.0	65.0 ± 2.9	119.0	0.548	158
#2	Control	53.4 ± 2.5	53.2 ± 5.0	113.0		112
	1 mM	53.5 ± 2.4	65.2 ± 3.3	113.0	0.649	105
	2 mM	51.0 ± 2.3	47.1 ± 4.0	113.0	0.349	114
#3	Control	61.4 ± 2.0	54.7 ± 2.7	103.0		116
	1 mM	55.2 ± 2.3	54.7 ± 3.1	103.0	0.191	126
	2 mM	59.2 ± 2.3	54.7 ± 3.3	103.0	0.894	112
Strain KU25 (*pmk-1* mutant)						
#1	Control	56.2 ± 1.5	52.3 ± 1.8	89.3		110
	1 mM	54.5 ± 1.3	52.3 ± 1.5	89.3	0.251	118
	2 mM	58.6 ± 1.9	52.3 ± 2.7	101.3	0.109	102
#2	Control	52.7 ± 2.0	53.2 ± 4.0	88.8		140
	1 mM	53.1 ± 1.9	53.2 ± 3.6	88.8	0.964	147
	2 mM	55.0 ± 1.8	53.2 ± 3.5	88.8	0.658	148
#3	Control	50.1 ± 2.3	42.5 ± 2.8	103.0		113
	1 mM	48.2 ± 2.2	54.7 ± 4.6	103.0	0.723	123
	2 mM	45.7 ± 2.0	42.5 ± 3.1	103.0	0.106	124

TABLE S7 List of differentially expressed genes. Differentially expressed genes (DEGs) regulated by AbaPep#07 in day 10 wild-type *C. elegans*. Related to Figure 5 and Figure 6.

See separate Excel file for details.

TABLE S8 References for SKN-1-targeted genes. Related to Figure 6b.

Number	Reference
R1	Oliveira, R. P., Porter Abate, J., Dilks, K., Landis, J., Ashraf, J., Murphy, C. T., & Blackwell, T. K. (2009). Condition-adapted stress and longevity gene regulation by *Caenorhabditis elegans* SKN-1/Nrf. *Aging Cell*, *8*(5), 524–541. https://doi.org/10.1111/j.1474-9726.2009.00501.x
R2	Rizki, G., Picard, C. L., Pereyra, C., & Lee, S. S. (2012). Host cell factor 1 inhibits SKN-1 to modulate oxidative stress responses in *Caenorhabditis elegans*. *Aging Cell*, *11*(4), 717–721. https://doi.org/10.1111/j.1474-9726.2012.00831.x
R3	Mark, K. A., Dumas, K. J., Bhaumik, D., Schilling, B., Davis, S., Oron, T. R., Sorensen, D. J., Lucanic, M., Brem, R. B., Melov, S., Ramanathan, A., Gibson, B. W., & Lithgow, G. J. (2016). Vitamin D promotes protein homeostasis and longevity via the stress response pathway genes *skn-1*, *ire-1*, and *xbp-1*. *Cell Reports*, *17*(5), 1227–1237. https://doi.org/10.1016/j.celrep.2016.09.086
R4	Nhan, J. D., Turner, C. D., Anderson, S. M., Yen, C. A., Dalton, H. M., Cheesman, H. K., Ruter, D. L., Uma Naresh, N., Haynes, C. M., Soukas, A. A., Pukkila-Worley, R., & Curran, S. P. (2019). Redirection of SKN-1 abates the negative metabolic outcomes of a perceived pathogen infection. *Proceedings of the National Academy of Sciences of the United States of America*, *116*(44), 22322–22330. https://doi.org/10.1073/pnas.1909666116
R5	Ewald, C. Y., Landis, J. N., Porter Abate, J., Murphy, C. T., & Blackwell, T. K. (2015). Dauer-independent insulin/IGF-1-signalling implicates collagen remodelling in longevity. *Nature*, *519*(7541), 97–101. https://doi.org/10.1038/nature14021
R6	Park, S. K., Tedesco, P. M., & Johnson, T. E. (2009). Oxidative stress and longevity in *Caenorhabditis elegans* as mediated by SKN-1. *Aging Cell*, *8*(3), 258–269. https://doi.org/10.1111/j.1474-9726.2009.00473.x
R7	Glover-Cutter, K. M., Lin, S., & Blackwell, T. K. (2013). Integration of the unfolded protein and oxidative stress responses through SKN-1/Nrf. *PLoS Genetics*, *9*(9), e1003701. https://doi.org/10.1371/journal.pgen.1003701
R8	Keshet, A., Mertenskötter, A., Winter, S. A., Brinkmann, V., Dölling, R., & Paul, R. J. (2017). PMK-1 p38 MAPK promotes cadmium stress resistance, the expression of SKN-1/Nrf and DAF-16 target genes, and protein biosynthesis in *Caenorhabditis elegans*. *Molecular Genetics and Genomics*, *292*(6), 1341–1361. https://doi.org/10.1007/s00438-017-1351-z

TABLE S9 List of primers used for quantitative real-time PCR analysis.

Gene	Primer type	Sequence (5'→3')	Reference
acdh-1	Forward	GCAAATGCAGATCCTAGCC	[1]
	Reverse	GTTTGTCTTCCTCCTTATCTACAG	
hphd-1	Forward	GATATTCCGAACAATGCCAG	[1]
	Reverse	TCCAAAGTCTCGCATATAACC	
pgp-5	Forward	ATCCGGTCTGTTGGACTCTTGG	[2]
	Reverse	TACCGTTCAACTTCTTGCTGCTG	
pgp-7	Forward	TGCTGCTACCGAAACGTCTATCC	[2]
	Reverse	CCAACGCTTCAATCGCAGTTC	
fmo-2	Forward	ATAATGAACACGCGTTTCTTC	[3]
	Reverse	GATGTTTGGCTTGATTCTGA	
cyp-35B2	Forward	TCTCGCAAGATCAGAATTGT	[4]
	Reverse	ACGATCCATGTGGTCTGATA	
ugt-26	Forward	AGGATTTCAGCTCAGTTCCTACTC	[5]
	Reverse	GGCGAGACTTGCTTTTGTTT	
ugt-29	Forward	TATATGCCAAAGAAATGGAGAAAC	[6]
	Reverse	CGAATACTGATAGTCGGGGATC	
irg-5	Forward	TCTTACTCTCCTGAACTTGTACC	[7]
	Reverse	TCGTACTTCTTCACCGCAG	
clec-265	Forward	AACTTTGCAGTTGGAGAACC	[8]
	Reverse	CTGAGCATACCAGTATCCGT	
act-1	Forward	CTCTTGCCCCATCAACCATG	[1]
	Reverse	CTTGCTTGGAGATCCACATC	

References for Table S9:

[1] Watson, E., Olin-Sandoval, V., Hoy, M. J., Li, C. H., Louisse, T., Yao, V., Mori, A., Holdorf, A. D., Troyanskaya, O. G., Ralser, M., & Walhout, A. J. (2016). Metabolic network rewiring of propionate flux compensates vitamin B12 deficiency in *C. elegans*. *eLife*, *5*, e17670. https://doi.org/10.7554/eLife.17670

[2] Guerrero, G. A., Derisbourg, M. J., Mayr, F. A., Wester, L. E., Giorda, M., Dinort, J. E., Hartman, M. D., Schilling, K., Alonso-De Gennaro, M. J., Lu, R. J., Benayoun, B. A., & Denzel, M. S. (2021). NHR-8 and P-glycoproteins uncouple xenobiotic resistance from longevity in chemosensory *C. elegans* mutants. *eLife*, *10*, e53174. https://doi.org/10.7554/eLife.53174

[3] Wani, K. A., Goswamy, D., Taubert, S., Ratnappan, R., Ghazi, A., & Irazoqui, J. E. (2021).

NHR-49/PPAR-α and HLH-30/TFEB cooperate for C. elegans host defense via a flavin-containing monooxygenase. *eLife*, *10*, e62775. https://doi.org/10.7554/eLife.62775

[4] Peltonen, J., Aarnio, V., Heikkinen, L., Lakso, M., & Wong, G. (2013). Chronic ethanol exposure increases cytochrome P-450 and decreases activated in blocked unfolded protein response gene family transcripts in *Caenorhabditis elegans*. *Journal of Biochemical and Molecular Toxicology*, *27*(3), 219–228. https://doi.org/10.1002/jbt.21473

[5] Kato, M., Kashem, M. A., & Cheng, C. (2016). An intestinal microRNA modulates the homeostatic adaptation to chronic oxidative stress in *C. elegans*. *Aging*, *8*(9), 1979–2005. https://doi.org/10.18632/aging.101029

[6] Wong, R. R., Kong, C., Lee, S. H., & Nathan, S. (2016). Detection of Burkholderia pseudomallei toxin-mediated inhibition of protein synthesis using a *Caenorhabditis elegans ugt-29* biosensor. *Scientific Reports*, *6*, 27475. https://doi.org/10.1038/srep27475

[7] Amrit, F. R. G., Naim, N., Ratnappan, R., Loose, J., Mason, C., Steenberge, L., McClendon, B. T., Wang, G., Driscoll, M., Yanowitz, J. L., & Ghazi, A. (2019). The longevity-promoting factor, TCER-1, widely represses stress resistance and innate immunity. *Nature Communications*, *10*(1), 3042. https://doi.org/10.1038/s41467-019-10759-z

[8] Wu, Z., Isik, M., Moroz, N., Steinbaugh, M. J., Zhang, P., & Blackwell, T. K. (2019). Dietary restriction extends lifespan through metabolic regulation of innate immunity. *Cell Metabolism*, *29*(5), 1192–1205.e8. https://doi.org/10.1016/j.cmet.2019.02.013
